# SmsB-SmsC machinery in *Thermococcus kodakarensis* functions as an archaeal scaffold mediating Fe-S cluster assembly

**DOI:** 10.1128/aem.01438-25

**Published:** 2025-08-18

**Authors:** Jian-qiang Jin, Takaaki Sato, Haruyuki Atomi

**Affiliations:** 1 Department of Synthetic Chemistry and Biological Chemistry, Graduate School of Engineering, Kyoto University 12918, Kyoto, Japan; 2 Integrated Research Center for Carbon Negative Science, Kyoto University 12918, Kyoto, Japan; University of Milano-Bicocca, Milano, Italy

**Keywords:** archaea, metabolism, iron-sulfur cluster assembly, SMS, SUF system, scaffold protein

## Abstract

The SUF system is one of the multiprotein machineries responsible for iron-sulfur (Fe-S) cluster biogenesis. In bacterial and eukaryotic SUF systems, SufB, SufC, and SufD form a SufBC_2_D complex as the scaffold for Fe-S cluster assembly. SMS (SUF-like minimal system), composed only of SufB and SufC homologs (SmsB and SmsC) without a SufD component, has recently been established, and representatives from methanogenic archaea have been experimentally verified to function in Fe-S cluster biogenesis. SMS has been proposed to be an ancestor of the SUF system. The hyperthermophilic archaeon *Thermococcus kodakarensis* harbors candidate proteins for SmsB and SmsC (*Tk*-SmsB and *Tk*-SmsC) encoded by TK0730 and TK0731 genes, respectively. As *Tk*-SmsB is phylogenetically positioned in a clade distinct from the previously characterized SmsB proteins from methanogens, here, we examined whether *Tk*-SmsB and *Tk*-SmsC can also function in Fe-S cluster generation and transfer. *Tk*-SmsB and *Tk*-SmsC formed a heterotetrameric SmsB_2_C_2_ complex. *Tk*-SmsC displayed ATPase activity, and its catalytic efficiency (*k*_cat_/*K*_m_) increased up to 10-fold upon complex formation with *Tk*-SmsB. The *Tk*-SmsB_2_C_2_ complex could generate Fe-S clusters in the presence of ferric and sulfide ions. The holo-*Tk*-SmsB_2_C_2_ complex could transfer the Fe-S clusters to the apo-form of the Fe-S cluster-dependent lipoyl synthase LipS from *T. kodakarensis*, resulting in a LipS with sulfur insertion activity for lipoyl group biosynthesis. In the presence of cysteine desulfurase and ATP, the apo-*Tk*-SmsB_2_C_2_ complex could also generate Fe-S clusters utilizing cysteine as a sulfur donor and activate LipS.

**IMPORTANCE:**

A representative of an SmsB protein from *T. kodakarensis* (*Tk*-SmsB) that lies in a clade phylogenetically distinct from those of previously verified SmsB proteins has been examined. The results demonstrate that *Tk*-SmsB, along with *Tk*-SmsC, functions as a scaffold for Fe-S cluster synthesis in *T. kodakarensis*, adding further support to the proposition that SMS represents the primitive form of the SUF systems widely present in bacteria and eukaryotes.

## INTRODUCTION

Iron-sulfur (Fe-S) clusters are versatile inorganic cofactors that are utilized for fundamental biological processes, such as nitrogen fixation, photosynthesis, respiration, and DNA repair (1–5). The well-known types of Fe-S clusters are [2Fe-2S] and [4Fe-4S] forms with rhombic and cubic structures, respectively (1, 6, 7). The Fe-S cluster assembly in living cells is a process controlled by several multiprotein machineries, the so-called Fe-S cluster biogenesis systems (8, 9). In the general principles of Fe-S cluster biogenesis (Fig. S1), a cysteine desulfurase (CSD) transfers sulfur from l-cysteine to a scaffold protein, which acts as a molecular platform for the assembly of iron and sulfur. The assembled Fe-S cluster is transferred to a target apoprotein directly or via carrier protein(s) to the target apoproteins. To date, three distinct systems responsible for Fe-S cluster assembly have been identified in bacteria, namely NIF (nitrogen fixation), ISC (iron sulfur cluster), and SUF (sulfur mobilization) systems (2, 3, 7–10).

The conventional SUF system is usually composed of cysteine desulfurase SufS, the scaffold proteins SufB and SufD, an ABC-type ATPase protein SufC, and the carrier proteins SufA or SufT. Other proteins that are involved include the SufS partner protein SufE or SufU, which enhances the SufS activity and/or is involved in sulfur transfer from SufS to SufB. Genes encoding the Suf proteins often form a *suf* operon on the genome (5, 11–13). The SUF system in *Escherichia coli* has been well studied and consists of SufA, SufB, SufC, SufD, SufE, and SufS proteins (14–23). In the *E. coli* system, the scaffold proteins SufB and SufD and the ABC-type ATPase protein, SufC, interact with each other to form a SufBC_2_D complex as the platform for Fe-S cluster assembly. This multi-protein scaffold is distinct from the single-protein scaffolds utilized in the ISC and NIF systems (5, 11, 13, 22, 24). SufD displays a relatively low identity to SufB (17% in *E. coli*). Nevertheless, SufB and SufD display structural similarity and share a common fold pattern (22). The Fe-S cluster is assembled at the heterodimer interface of SufB and SufD. SufC contains motifs that are characteristic of ATP-binding cassette (ABC)-type ATPases, such as the Walker A and B motifs, D- and Q-loops, as well as the ABC signature (5, 25–27). The ATPase activity of SufC is significantly enhanced upon interaction with SufB or SufD (28, 29). The exact role of the ATPase activity of SufC in the SufBC_2_D complex is still unclear. In addition, *in vitro* analysis showed that SufC was able to interact solely with SufB or SufD, forming a SufB_2_C_2_ complex or SufCD/SufC_2_D_2_ complexes, respectively (20, 28, 30, 31).

SUF systems have been studied in various bacteria and eukaryotes, such as *Thermus thermophilus* (29), *Thermotoga maritima* (28, 32), *Arabidopsis thaliana* (33, 34), and *Plasmodium falciparum* (35). Archaea species were predicted to possess only SUF systems for Fe-S cluster biogenesis, since the homologs for ISC and NIF systems were not found. However, the major SUF components, including SufA/SufT, the partner protein SufE/SufU, and in many cases SufD, are missing in most archaeal members (3, 11, 24, 36). Recently, Garcia et al. reported two minimal Fe-S cluster assembly systems called MIS (minimal iron-sulfur) and SMS (SUF-like minimal system) in the methanogenic archaea *Methanosarcina acetivorans* and *Methanocaldococcus jannaschii* (37). The MIS system is composed of a pair of homologs for IscS (a cysteine desulfurase of the ISC system) and IscU (a scaffold protein of the ISC system), which were named MisS and MisU, respectively. Members of the SMS system are two homologs of the SUF scaffold proteins SufB and SufC, which were named SmsB and SmsC. The SmsB and SmsC proteins from *M. acetivorans* and *M. jannaschii* were validated to function as the Fe-S cluster assembly scaffold, and their gene homologs, widely distributed in *Archaea*, were designated as Sms proteins (37).

In the hyperthermophilic archaeon *Thermococcus kodakarensis*, candidates for the *smsB* (TK0730) and *smsC* (TK0731) genes are present on the genome. *T. kodakarensis* also possesses a gene (TK1990) encoding CSD and a gene (TK1693) encoding a protein displaying 20% identity with the characterized SufT protein from *Staphylococcus aureus* (38). The cysteine desulfurase activity of the TK1990 protein has been verified in a previous study (39). In this study, we characterized the SmsB and SmsC candidates and examined their involvement in Fe-S cluster assembly in *T. kodakarensis*. We show that the TK0730 protein (*Tk*-SmsB) and the TK0731 protein (*Tk*-SmsC) form a SmsB_2_C_2_ complex as the functional platform for Fe-S cluster assembly.

## RESULTS

### Structural characteristics of the SmsB and SmsC homologs in *T. kodakarensis*

*Tk*-SmsB and *Tk*-SmsC are encoded by the TK0730 and TK0731 genes, respectively, which are predicted to form an operon based on previous transcriptome studies (40). *Tk*-SmsC resembles the bacterial SufC proteins from *E. coli* (46% identity), *B. subtilis* (42%), *T. thermophilus* (44%), and *T. maritima* (51%), and the SmsC from *M. acetivorans* (36%) and *M. jannaschii* (39%). *Tk*-SmsC harbors the conserved Walker A and B motifs, D- and Q-loops, and ABC signature motif, all of which are characteristic of ABC ATPases (Fig. S2A) (22, 26). *Tk*-SmsB shares relatively lower identities with the bacterial SufB proteins from *E. coli* (*Ec*-SufB) (22%), *B. subtilis* (31%), *T. thermophilus* (30%), and *T. maritima* (32%). Identities with the eukaryotic SufBs and the methanogenic SmsBs are also lower—SufBs from *A. thaliana* (21%) and *P. falciparum* (19%), and SmsBs from *M. acetivorans* (27%) and *M. jannaschii* (18%).

The structure of the SufBC_2_D complex from *E. coli* has been studied (22, 41). Most of the critical residues responsible for sulfur transfer and Fe-S cluster coordination are conserved in *Tk*-SmsB (Fig. S2B and C). They include the residues C357, H385, and E386 in *Tk*-SmsB corresponding to the residues C405 in *Ec*-SufB, H360 in *Ec*-SufD, and E434 in *Ec*-SufB, respectively, which are located at the interface between *Ec*-SufB and *Ec*-SufD and coordinate the Fe-S cluster. Since each *Tk*-SmsB has three residues that could serve as ligands, the *Tk*-SmsB_2_C_2_ complex should be able to provide enough ligand residues to coordinate an Fe-S cluster.

### Phylogenetic analysis of archaeal SufB, SmsB, and SufD homologs

**Fig 1:**
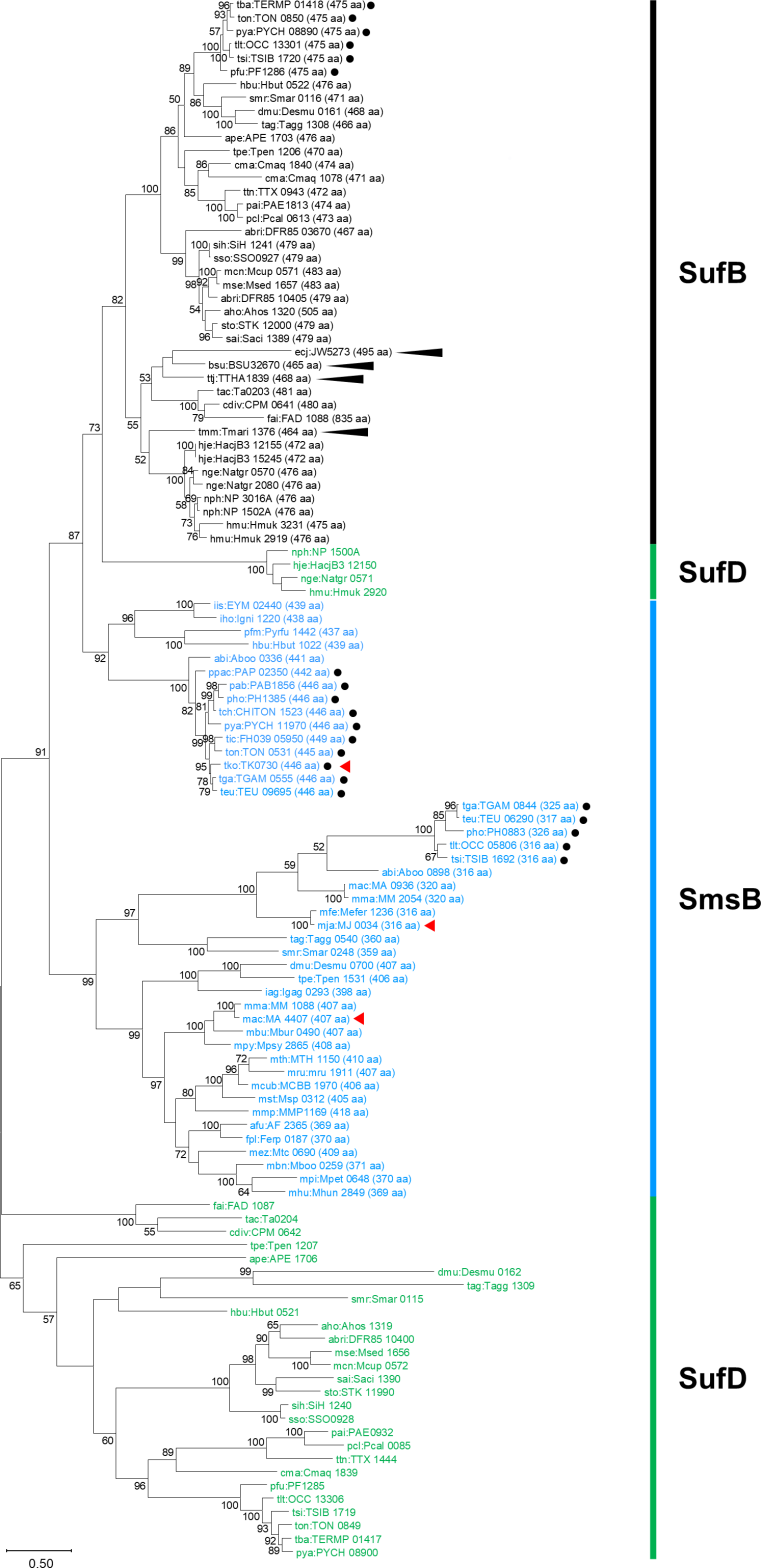
Phylogenetic analysis of SmsB, SufB, and SufD homologs in selected species of *Archaea*. The archaeal SmsB, SufB, and SufD homologs (113 sequences from 61 organisms, listed in Table S1) and four bacterial SufBs (indicated by black elongated arrowheads) were analyzed. The sequence lengths of SufB and SmsB homologs are added in parentheses following their locus tags. The SufB and SmsB homologs from *Thermococcales* are indicated by black dots. The SufB, SmsB, and SufD groups are indicated by black, blue, and green bars, respectively. *Tk*-SmsB and previously characterized methanogenic SmsBs are indicated by red arrowheads. Organism codes are shown in Table S1. Bootstrap values higher than 50% are shown. The phylogenetic tree was constructed by MEGA12. How to distinguish SmsB and SufB was based on the definition proposed in the previous report (37).

A phylogenetic analysis of SufB, SmsB, and SufD homologs selected from archaea and bacteria (listed in Table S1) indicates that the SufB homologs cluster in one group (Fig. 1). Archaea that harbor SufB homologs also harbor SufC and SufD homologs, and the genes tend to form *sufBCD* operons. One can also observe that the archaeal SmsB proteins lie in two clades; one of which includes the proteins from methanogens that have been demonstrated to function as components of SMS. This clade includes members from methanogens and some species of *Archaeoglobales*, *Thermococcales*, *Desulfurococcales*, and *Thermofilales*. The other clade is more related to the SufB homologs and includes *Tk*-SmsB and proteins from other members of *Thermococcales* and *Desulfurococcales*. As no member from the latter clade has been experimentally verified, we examined whether *Tk*-SmsB can function as a scaffold protein for Fe-S generation in *T. kodakarensis*.

### Purification of the recombinant TK0730, TK0731, and TK1990 proteins

The recombinant *Tk*-SmsB (with a 6× His tag at its N-terminus) and *Tk*-SmsC proteins were purified. Judging from the results of sodium dodecyl sulfate-polyacrylamide gel electrophoresis (SDS-PAGE), these proteins were purified to apparent homogeneity (Fig. S3A and B). In order to examine CSD-based Fe-S cluster assembly *in vitro*, the previously characterized CSD of *T. kodakarensis* (TK1990; *Tk*-CSD) (39) was also prepared using *E. coli* and purified (Fig. S3C).

### Interaction between the *Tk*-SmsB and *Tk*-SmsC proteins

**Fig 2:**
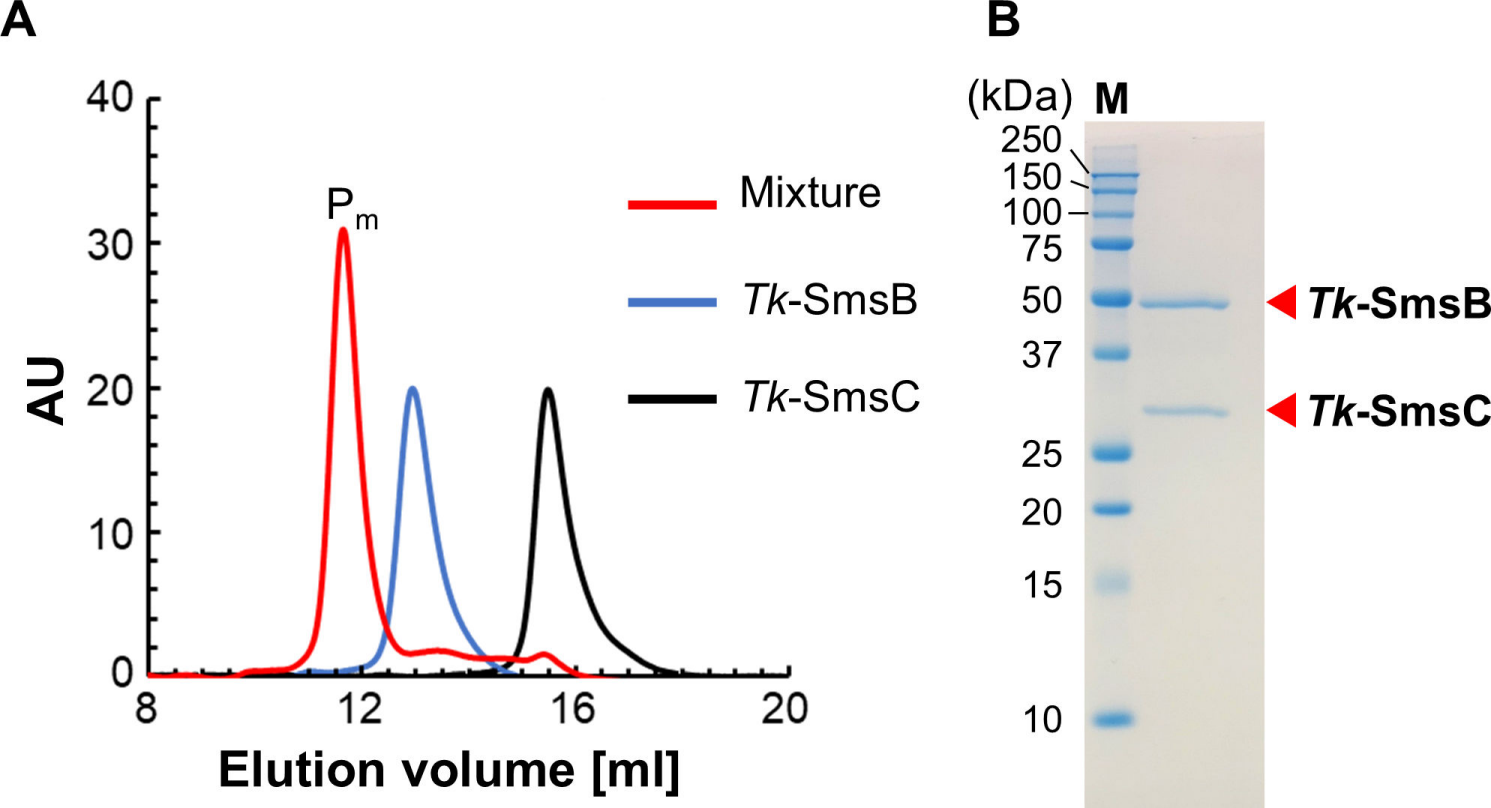
Association analysis of *Tk*-SmsB and *Tk*-SmsC proteins. (**A**) Chromatogram of gel filtration chromatography analyzing *Tk*-SmsB protein (40 µM), *Tk*-SmsC protein (72 µM), and their mixture. A Superdex 200 Increase 10/300 Gl column was used. The mixture was composed of equimolar *Tk*-SmsB protein and *Tk*-SmsC protein (40 µM each). AU indicates arbitrary units. (**B**) SDS-PAGE analysis of a fraction corresponding to the peak P_m_ in the protein mixture solution (11.5 to 12.0 mL). M indicates the molecular mass marker.

Association analysis of *Tk*-SmsB and *Tk*-SmsC proteins was performed by gel filtration chromatography (Fig. 2A). The elution volume of the *Tk*-SmsB protein alone (40 µM) corresponded to a molecular mass of 95.4 kDa, approximately 2-fold of the theoretical molecular mass of the monomer (50.8 kDa, including His-tag molecular mass), suggesting that the *Tk*-SmsB protein alone in solution forms a homodimer. The elution volume of *Tk*-SmsC protein alone (72 µM) corresponded to a molecular mass of 28.7 kDa. The theoretical molecular mass of *Tk*-SmsC is 27.5 kDa, suggesting that the protein is monomeric in solution. The major peak (P_m_) of an equimolar mixture of the *Tk*-SmsB and *Tk*-SmsC proteins (each 40 µM) eluted at 11.7 mL, corresponding to a molecular mass of 164.6 kDa. The result raised the possibility that these two proteins form a heterotetrameric complex in the mixture with a 2:2 stoichiometry (theoretical molecular mass of 156.7 kDa), corresponding to a SmsB_2_C_2_ complex. A fraction including the P_m_ peak (from 11.5 to 12.0 mL) was analyzed by SDS-PAGE (Fig. 2B). Two bands whose molecular masses corresponded to those of the *Tk*-SmsB protein and *Tk*-SmsC protein were observed, further supporting that the two proteins form a complex. We also measured the densities of these bands, and the density ratio of *Tk*-SmsB to *Tk*-SmsC was 1.13. The relative densities of the bands also do not disagree with a 2:2 stoichiometry.

### Pull-down assay to examine the presence of a potential SufD-like protein in *T. kodakarensis*

Although a SufD homolog could not be found on the genome of *T. kodakarensis*, we could not exclude the possibility of proteins other than *Tk*-SmsB and *Tk*-SmsC participating in the formation of the scaffold. To address this, we carried out a protein pull-down assay taking advantage of the His-tag in *Tk*-SmsB. Assays were performed using the *Tk*-SmsB recombinant protein as bait against the cell-free extract of *T. kodakarensis* (Fig. S4). *Tk*-SmsC was clearly observed in the pull-down fraction, adding strong support that these proteins form a complex *in vivo* (Lane E3 in Fig. S4). In addition to the *Tk*-SmsC protein, several other proteins eluted together with *Tk*-SmsB or the *Tk*-SmsCB complex. However, these proteins were also observed in the negative controls with similar abundance (Lane E1 and E2 in Fig. S4), suggesting that they are not interacting specifically with *Tk*-SmsB or the *Tk*-SmsCB complex. Although recombinant *Tk*-SmsC was further added into the mixture to increase the amount of SmsCB complex, the result was similar (E4 in Fig. S4). Although we cannot exclude the possibility that the His-tag on the SmsB recombinant protein hampers interaction with other proteins, the result of the pull-down assay suggests that there is no other protein that binds to SmsB or its complex with SmsC, such as the SufD in the scaffold from *E. coli*. Together with the results of gel-filtration chromatography, the result suggests that *T. kodakarensis* employs a SmsB_2_C_2_ complex for Fe-S cluster assembly.

### ATPase activity measurement of *Tk*-SmsC protein

**Fig 3:**
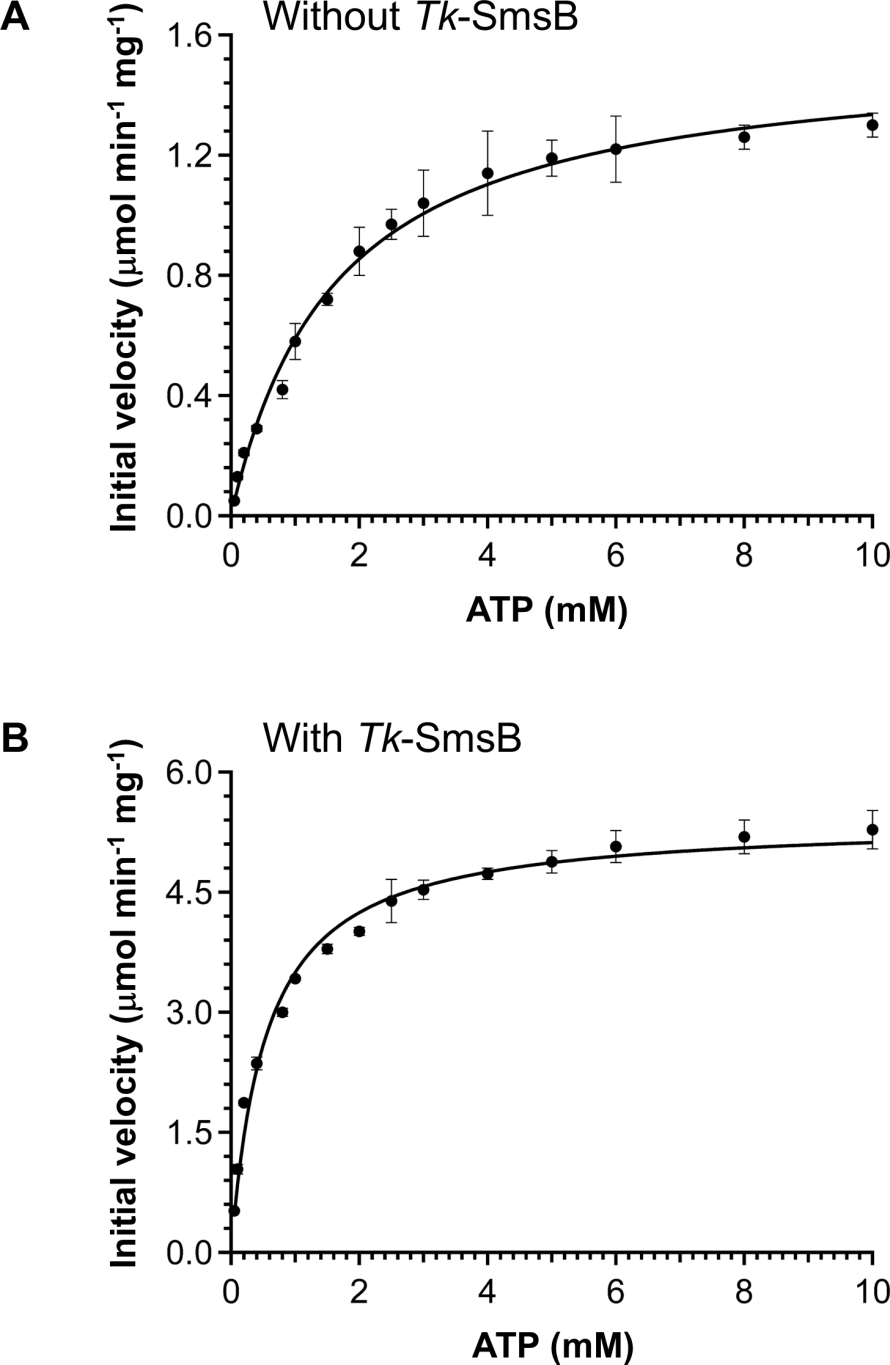
Kinetic analysis of the ATP hydrolysis catalyzed by *Tk*-SmsC protein. (**A**) The assay was carried out with 50 mM HEPES (pH 7.5), 5 mM MgCl_2_, and *Tk*-SmsC protein (0.3 or 0.6 µM) at 75°C. (**B**) *Tk*-SmsB protein (equal molar concentration) was further added to the reaction mixture. Error bars indicate the standard deviations of three independent experiments.

The hydrolysis of ATP catalyzed by the recombinant *Tk*-SmsC protein was examined. *Tk*-SmsC alone catalyzed ATP hydrolysis and displayed a specific activity of 1.5 µmol min^−1^ mg^−1^. This activity increased to 4.6 µmol min^−1^ mg^−1^ in the presence of an equimolar concentration of *Tk*-SmsB. This is similar to the ATPase activity of bacterial SufC, which is enhanced upon interacting with SufB (28). Kinetic analysis showed that the *V*_max_ values of *Tk*-SmsC protein alone and the *Tk*-SmsB_2_C_2_ complex were 1.6 and 5.4 µmol min^−1^ mg^−1^, respectively (Fig. 3; Table 1). The *K*_m_ value of *Tk*-SmsC toward ATP was reduced (about 67% decrease) upon interaction with the *Tk*-SmsB protein, suggesting that *Tk*-SmsB mediates a conformational change in *Tk*-SmsC to better recognize ATP. The catalytic efficiency (*k*_cat_/*K*_m_) of *Tk*-SmsC was significantly increased (approximately 10-fold) by forming a complex with *Tk*-SmsB (Table 1).

**TABLE 1:** Kinetic parameters of the *Tk*-SmsC protein

	*V*_max_ (μmol min^−1^ mg^−1^)	*K*_m_ (mM)	*k*_cat_ (s^−1^)	*k*_cat_/*K*_m_ (M^−1^ s^−1^)
*Tk*-SmsC	1.6 ± 0.0	1.6 ± 0.1	0.7 ± 0.0	4.4 × 10^2^
*Tk*-SmsB_2_C_2_	5.4 ± 0.1	0.5 ± 0.0	2.5 ± 0.0	4.6 × 10^3^

### Examination of *Tk*-CSD

CSDs can be classified into two types based on their primary structures: type I includes NifS, IscS, and MisS, which belong to the NIF, ISC, and MIS systems, respectively, whereas type II includes SufS of the SUF system (42, 43). *T. kodakarensis* possesses a sole CSD homolog, TK1990, encoding *Tk*-CSD (Table S1). *Tk*-CSD shares a higher identity with SufS from *E. coli* (37.5%) than with IscS from *E. coli* (20.8%), NifS from *Azotobacter vinelandii* (18.0%), and MisS from *M. acetivorans* (22.5%), suggesting that *Tk*-CSD may be involved in Fe-S cluster generation on the *Tk*-SmsB_2_C_2_ complexes.

We examined the interaction between *Tk*-CSD and *Tk*-SmsB/SmsC. *Tk*-CSD itself was eluted at around 13.7 mL (Fig. S5A, yellow line), corresponding to a molecular mass of 86.6 kDa, approximately 2-fold of the theoretical molecular mass of the monomer (44.4 kDa), suggesting that the *Tk*-CSD protein alone in solution forms a homodimer. We confirmed that *Tk*-CSD interacts with neither *Tk*-SmsC nor *Tk*-SmsB_2_C_2_ complex (Fig. S5A and B). It was difficult to confirm whether *Tk*-CSD forms a heterodimer complex with *Tk*-SmsB based on the gel filtration chromatography, as they themselves form a homodimer and display similar molecular masses (Fig. S5A, blue line). However, the amounts of *Tk*-CSD and *Tk*-SmsB were not equal in each eluted fraction of *Tk*-CSD-SmsB mixture (Fig. S5B, Lanes 1-5), suggesting that these two proteins did not form a complex. To further confirm this, we carried out affinity chromatography and found that *Tk*-CSD was not eluted together with *Tk*-SmsB (Fig. S5C). These results suggest that *Tk*-CSD does not form a complex with *Tk*-SmsB/SmsC, at least under the applied conditions.

### *In vitro* examination of Fe-S cluster assembly on the *Tk*-SmsB_2_C_2_ complex

**Fig 4:**
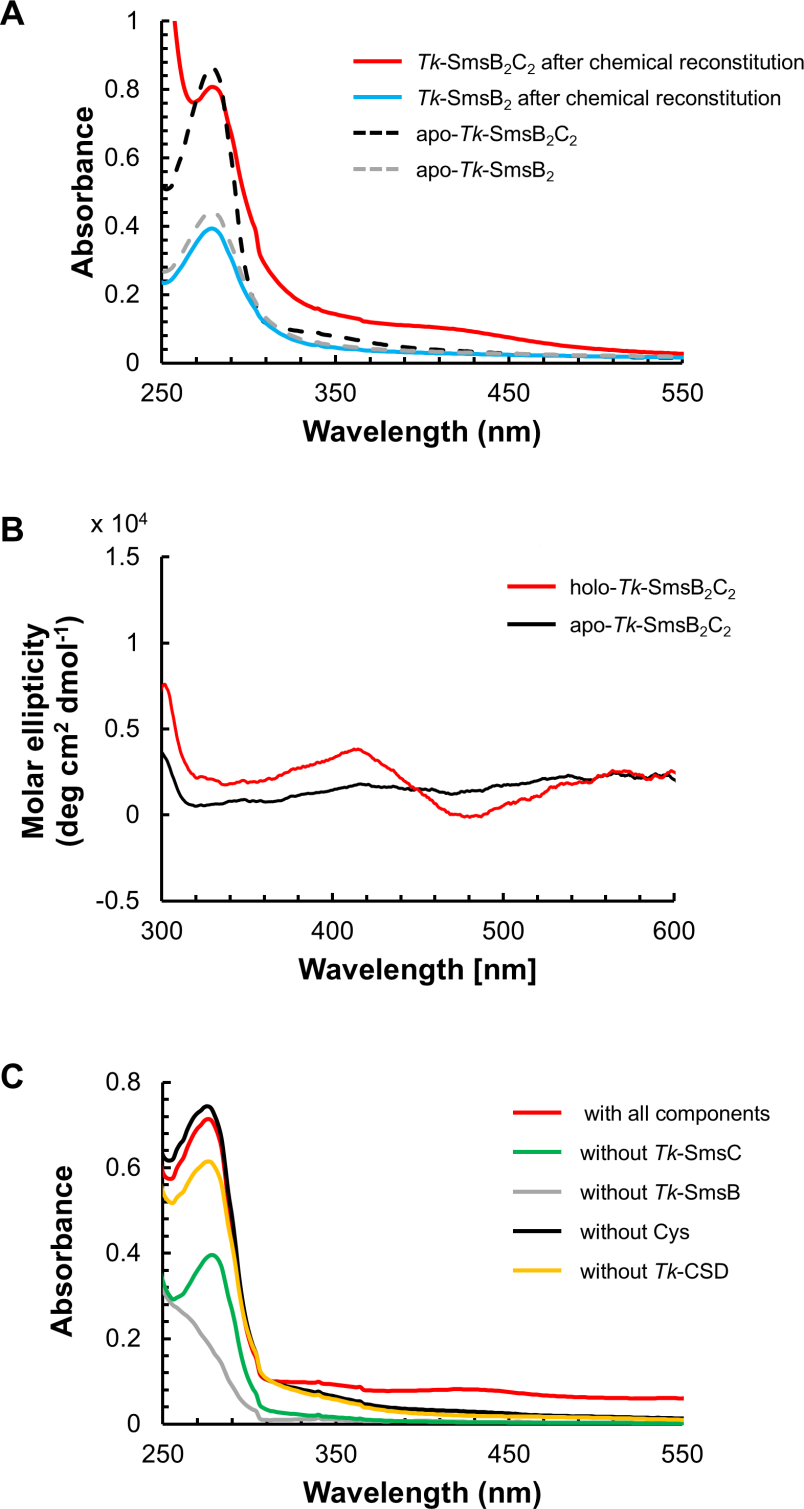
UV-visible and circular dichroism spectra of Fe-S clusters on *Tk*-SmsB_2_C_2_ complex. (**A**) UV-visible spectra of Fe-S clusters on *Tk*-SmsB protein (20 µM) and *Tk*-SmsB_2_C_2_ complex (10 µM) formed by chemical reconstitution. Gray-dashed line, *Tk*-SmsB protein before reconstitution; black-dashed line, *Tk*-SmsB_2_C_2_ complex before reconstitution; blue line, *Tk*-SmsB protein after reconstitution; and red line, *Tk*-SmsB_2_C_2_ complex after reconstitution. (**B**) Circular dichroism spectra of Fe-S clusters on *Tk*-SmsB_2_C_2_ complex (100 µM). The apo-form (black line) and chemically reconstituted (red line) *Tk*-SmsB_2_C_2_ complexes were monitored. (**C**) Fe-S clusters on *Tk*-SmsB_2_C_2_ scaffold complex (10 µM) formed by CSD-based reconstitution. Black line, protein mixture (*Tk*-SmsB, *Tk*-SmsC, and *Tk*-CSD) without Cys; gray line, protein mixture without *Tk*-SmsB; green line, protein mixture without *Tk*-SmsC; yellow line, protein mixture without *Tk*-CSD; and red line, protein mixture with all components.

We examined whether the SmsB_2_C_2_ complex of *T. kodakarensis* could function for Fe-S cluster assembly without additional components. The apo-form of *Tk*-SmsB was used as the substrate to examine chemical reconstitution of the Fe-S cluster in the presence or absence of *Tk*-SmsC. The UV-visible spectra of proteins with or without treatment by free ferric and sulfide ions were measured (Fig. 4A). Although measures were taken to remove iron-sulfide not associated with the protein, their presence in the sample can be expected. Compared with the sample without the reconstitution treatment (apo-form), *Tk*-SmsB_2_C_2_ complex after the reconstitution procedure exhibited a broad absorption band at around 420 nm, which is considered a UV-visible spectrum characteristic of [4Fe-4S] clusters (19–21). Absorbance at 280 nm of apo- and holo-complexes suggests that the amounts of proteins were at similar levels. To further confirm the presence of [4Fe-4S] cluster on the complex, we carried out circular dichroism (CD) spectroscopy analysis and observed a CD spectrum, suggesting the presence of proteins with [4Fe-4S] clusters (Fig. 4B). Quantitative measurement showed that 1 nmol of reconstituted *Tk*-SmsB_2_C_2_ complex contained 3.35 ± 0.08 nmol of iron atoms and 3.15 ± 0.17 nmol of sulfur atoms, suggesting that a [4Fe-4S] cluster is formed. In contrast, *Tk*-SmsB alone after reconstitution did not display this absorbance at 420 nm (Fig. 4A). As described above, the Fe-S cluster assembly site at the interface of the SufB-SufD heterodimer in the SufBC_2_D complex is buried in the resting state. As *Tk*-SmsB itself is considered to form a homodimer in solution, the assembly site may also be buried, and thus, ferric and sulfide ions would not be able to access their binding site in the *Tk*-SmsB protein when *Tk*-SmsC is absent. On the other hand, the presence or absence of ATP and Mg^2+^ ions, required for ATPase activity, did not affect the assembly of an Fe-S cluster when performing the chemical reconstitution (Fig. S6A). If the assembly site is located at the interface of the *Tk*-SmsB homodimer, this result suggests that the assembly site is exposed upon *Tk*-SmsB binding with *Tk*-SmsC, irrespective of the presence or absence of ATP.

We next pursued CSD-based reconstitution of a [4Fe-4S] cluster on the *Tk*-SmsB_2_C_2_ complex. In this experiment, sulfur atoms for Fe-S cluster assembly originate from cysteine, which undergoes desulfurization catalyzed by *Tk*-CSD. An absorption peak suggesting the presence of a [4Fe-4S] cluster was observed in the reaction mixture with all components (Fig. 4C), whereas those without SmsB, SmsC, *Tk*-CSD, or cysteine did not show the characteristic spectrum. The concentrations of *Tk*-SmsB and *Tk*-SmsC in the reaction mixture were the same as those in the reaction mixture of chemical reconstitution (Fig. 4A), and comparable levels of Fe-S cluster assembly were observed. These results indicated that although we could not detect complex formation, *Tk*-CSD can function in Fe-S cluster generation on the *Tk*-SmsB_2_C_2_ complex *in vitro*. Further analysis will be necessary to understand the *in vivo* specificity of *Tk*-CSD. In addition, ATP was required for the CSD-dependent [4Fe-4S] cluster generation of *Tk*-SmsB_2_C_2_ complex (Fig. S6B), but the mechanism is unclear.

### Examination of the Fe-S cluster transfer from holo-*Tk*-SmsB_2_C_2_ to apo-LipS

**Fig 5:**
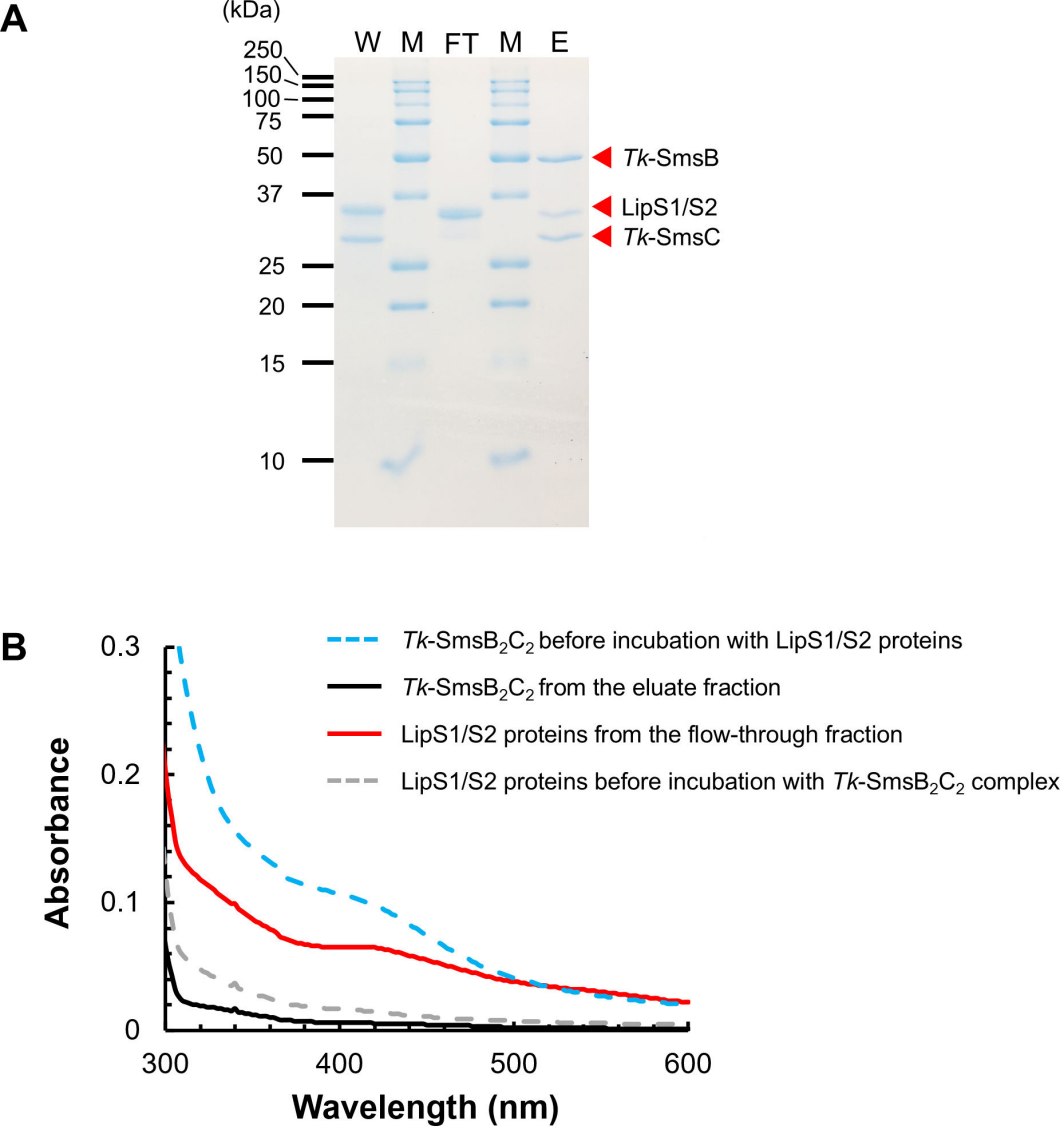
Detection of Fe-S cluster transfer. (**A**) SDS-PAGE analysis of the proteins in various fractions after separation with a His GraviTrap column. Abbreviations: M, molecular mass marker; FT, flow-through fraction; W, wash fraction; E, eluate fraction. (**B**) UV-visible spectra of Fe-S clusters on the *Tk*-SmsB_2_C_2_ complex and LipS1/S2 before and after the transfer reaction. Blue-dashed line, the holo-*Tk*-SmsB_2_C_2_ complex before incubation with LipS1/S2 proteins; gray-dashed line, LipS1/S2 proteins before incubation with the holo-*Tk*-SmsB_2_C_2_ complex; black line, *Tk*-SmsB_2_C_2_ complex from the eluate fraction; and red line, LipS1/S2 proteins from the flow-through fraction.

In previous studies, a novel lipoyl synthase, LipS, was identified in *T. kodakarensis*. LipS was shown to be a [4Fe-4S] cluster-dependent enzyme, composed of two separate proteins, LipS1 and LipS2 (44, 45). To examine the transfer of a [4Fe-4S] cluster to LipS1/S2, the LipS1/S2 apoprotein was incubated with the holo-*Tk*-SmsB_2_C_2_ complex on which the Fe-S cluster was chemically reconstituted. After incubation, the proteins were concentrated and subjected to nickel affinity chromatography, through which the *Tk*-SmsB_2_C_2_ complex (*Tk*-SmsB containing His-tag) and LipS1/S2 proteins (without His-tag) could be separated. The flow-through and wash fractions displayed a brown color, whereas the eluate fraction did not. SDS-PAGE analysis showed that the LipS1/S2 proteins were mainly present in the flow-through and wash fractions (Fig. 5A). LipS1/S2 proteins from the flow-through fraction displayed the absorption band suggesting a [4Fe-4S] cluster (Fig. 5B, red line), whereas LipS1/S2 proteins prior to incubation with the *Tk*-SmsB_2_C_2_ complex did not (Fig. 5B, gray-dashed line). These results suggested the presence of a [4Fe-4S] cluster on LipS1/S2 proteins after incubation with holo-*Tk*-SmsB_2_C_2_. On the other hand, *Tk*-SmsB_2_C_2_ complex, which initially exhibited absorbance at around 420 nm (Fig. 5B, blue-dashed line), lost the absorption peak after incubation with LipS1/S2 proteins (Fig. 5B, black line). The results strongly suggest that an Fe-S cluster transfer occurred from holo-*Tk*-SmsB_2_C_2_ to apo-LipS1/S2 proteins.

### Examination of LipS activity with *Tk*-SmsB_2_C_2_ complex

**Fig 6:**
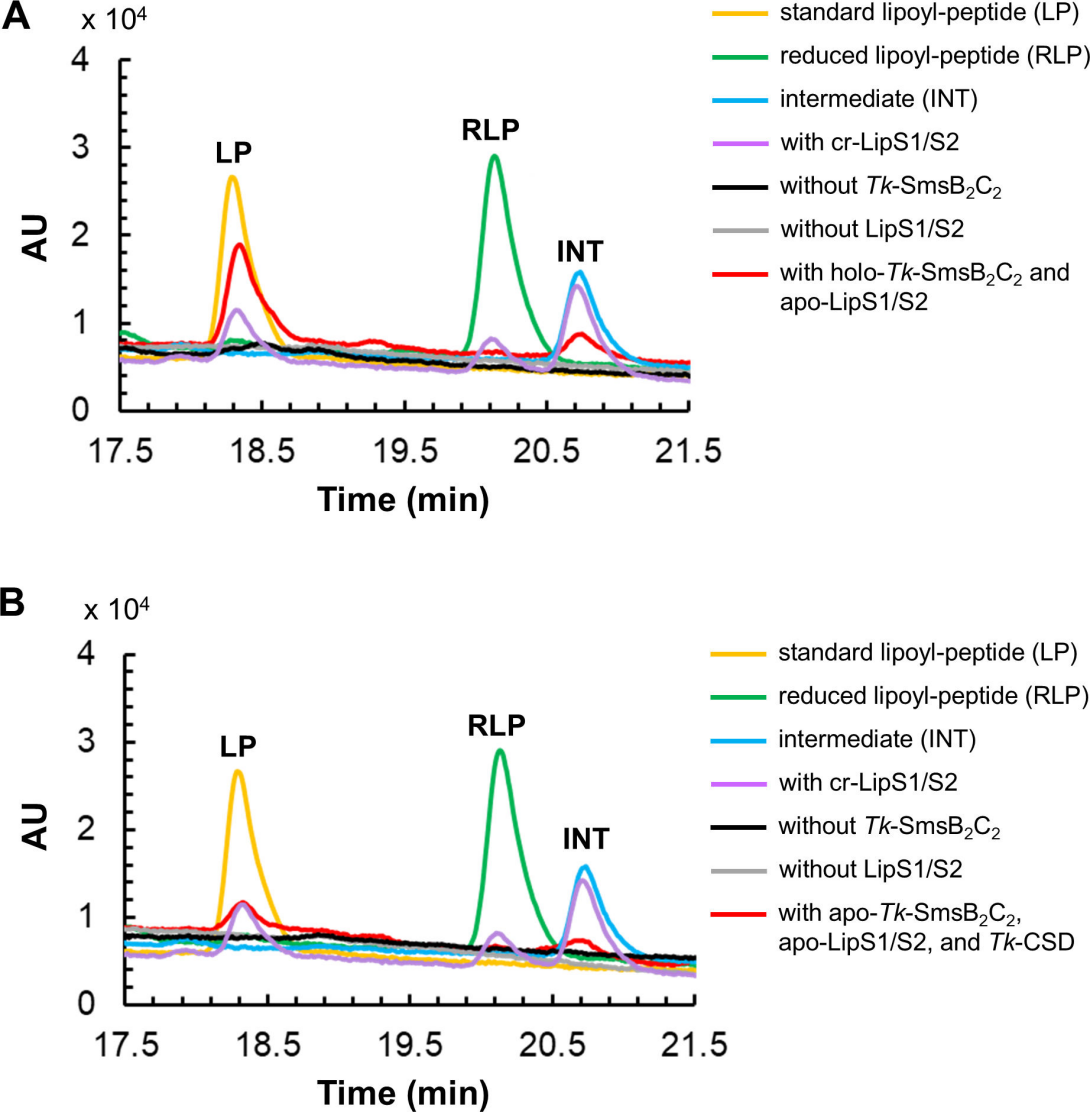
Lipoyl synthase activity measurement by HPLC analysis. (**A**) Lipoyl synthase reaction catalyzed by LipS1/S2 proteins in the presence or absence of holo-*Tk*-SmsB_2_C_2_. Reaction product without holo-*Tk*-SmsB_2_C_2_ (black line), reaction product without LipS1/S2 proteins (gray line), and reaction product with LipS1/S2 proteins and holo-*Tk*-SmsB_2_C_2_ complex (red line) were analyzed. (**B**) Lipoyl synthase reaction catalyzed by LipS1/S2 proteins in the presence of *Tk*-SmsB_2_C_2_ complex and *Tk*-CSD protein. Reaction product without *Tk*-SmsB_2_C_2_ complex (black line), reaction product without LipS1/S2 proteins (gray line), and reaction product with LipS1/S2 proteins, *Tk*-SmsB_2_C_2_ complex, and *Tk*-CSD protein (red line) were analyzed. Yellow line, the standard product lipoyl-peptide; green line, the reduced form of lipoyl-peptide prepared by treatment with DTT; blue line, the intermediate prepared by LipS2 reaction; and purple line, the reaction product catalyzed by LipS1/S2 in which the Fe-S cluster was chemically reconstituted (cr-LipS1/S2).

Since the spectroscopic data suggested that an Fe-S cluster was transferred from holo-*Tk*-SmsB_2_C_2_ to apo-LipS1/S2 proteins, the lipoyl synthase activity of LipS1/S2 was examined. The lipoyl synthase reaction was carried out by incubating apo-LipS1/S2, the chemically reconstituted holo-*Tk*-SmsB_2_C_2_ complex, and octanoyl-peptide substrate. In the reaction mixture, apo-LipS1 and apo-LipS2 were present at a concentration of 50 µM each along with 150 µM of the substrate octanoyl-peptide. To provide holo-*Tk*-SmsB_2_C_2_, *Tk*-SmsB, and *Tk*-SmsC were chemically reconstituted and added to the reaction mixture at a final concentration of 200 µM for each polypeptide. The reaction products were analyzed by HPLC (Fig. 6A). The standard product lipoyl-peptide and its reduced form (reduced lipoyl-peptide) were detected at 18.3 and 20.1 min, respectively. The intermediate thiol-octanoyl-peptide, which was prepared by using chemically reconstituted LipS2 protein alone (44), eluted at 20.7 min. Products generated by chemically reconstituted holo-LipS1/S2 were also analyzed as a positive control. After the reaction, a peak whose retention time corresponded to that of lipoyl-peptide was observed in the reaction mixture with holo-*Tk*-SmsB_2_C_2_ and apo-LipS1/S2 (Fig. 6A, red line). The concentration of the generated product lipoyl-peptide after 30 min was approximately 14.5 µM, indicating that at least 29 µM of Fe-S clusters providing the sulfur atoms for insertion into the octanoyl group were transferred from holo-*Tk*-SmsB_2_C_2_ complex to apo-LipS1/LipS2. In addition, another peak with the same retention time as the intermediate (thiol-octanoyl-peptide) was also observed. In contrast, production of lipoyl-peptide was not detected in the reaction mixtures without apo-LipS1/S2 or holo-*Tk*-SmsB_2_C_2_. Furthermore, the lipoyl synthase activity of apo-LipS1/S2 incubated with apo-*Tk*-SmsB_2_C_2_ and *Tk*-CSD was also examined in the presence of Cys (Fig. 6B). As in the case using holo-*Tk*-SmsB_2_C_2_, two peaks corresponding to the product lipoyl-peptide and the intermediate were detected in the reaction mixture containing all proteins (apo-LipS1/S2, apo-*Tk*-SmsB_2_C_2_, and *Tk*-CSD). These results strongly suggest that *Tk*-SmsB_2_C_2_ can act as a scaffold for Fe-S cluster generation using sulfide provided by CSD from cysteine, and also activate apo-LipS1/S2 to function in sulfur insertion, most likely by transferring [4Fe-4S] clusters.

## DISCUSSION

The SufBC_2_D complex is the scaffold of Fe-S cluster assembly in bacterial and eukaryotic SUF systems. This is not universal in archaea, as demonstrated with SMS from methanogens (37). Our results add support that the archaeal SmsB_2_C_2_ complexes are capable of Fe-S cluster assembly and transfer.

Differences in primary structure can be observed among the SmsB proteins (Fig. S7). The N-terminal regions of SmsBs in the clade, including those from methanogens, are much shorter than those in the clade including *Tk*-SmsB and SufBs (Fig. S7A). Consequently, the lengths of the entire sequences of SufBs and SmsBs tend to differ: SufBs consist of more than 460 residues; the majority of SmsBs are less than 420 residues; and the SmsBs similar to *Tk*-SmsB seem to consist of 430–450 residues. Primary sequences in the N-terminal regions of SmsB in the subgroup are much more similar to those of SufBs compared with the majority of SmsBs (Fig. S7B and C). On the other hand, SufB and SmsB proteins display similarity in the middle and C-terminal sections, especially the key residues that play roles in sulfur transfer and cluster ligands that are highly conserved (Fig. S7A). The 3D-structural comparison of *Tk*-SmsB with SmsB from *M. jannaschii* (Fig. S8) also showed that they are highly similar in the middle and C-terminal sections, only displaying differences in the N-termini. As the SMS has been proposed to be the ancestor of the current SUF system (37), the divergence of N-terminal regions in SmsB members may also reflect the evolutionary route of SufB.

A previous study demonstrated that the methanogenic archaeon *Methanococcus maripaludis*, which lacks the SufS homolog, actually utilizes exogenous sulfide ions as a sulfur source for Fe-S cluster assembly instead of l-cysteine, likely due to the availability of abundant sulfide in the environment it inhabits (46). Although *T. kodakarensis* only harbors a single SufS homolog TK1990 (Table S1), it is not located in the *suf* operon. It has been demonstrated that TK1990 is not essential for *T. kodakarensis* when elemental sulfur (S^0^) is available, since this organism could utilize S^0^ as the original source of sulfur atoms for Fe-S cluster assembly (39). The addition of polysulfide could also rescue the growth defect. However, sulfide could not be utilized as an alternative sulfur source. The study suggests that *T. kodakarensis* possesses two routes to provide sulfur atoms for the biogenesis of Fe-S clusters, an S^0^-dependent pathway and a CSD-dependent pathway. Although it is unclear how the TK1990 disruption strain can generate Fe-S clusters without a cysteine desulfurase, the results obtained here imply that the intermediate of the alternative route, dependent on S^0^, is a sulfide ion originating from S^0^. The observation that the addition of sulfide could not complement the growth of a mutant lacking CSD may be attributed to the lack of a sulfide transport system in *T. kodakarensis*. The S^0^-dependent pathway in *T. kodakarensis* can be regarded as a simpler way for Fe-S cluster assembly compared with those in bacteria and eukaryotes, which require SufS, SufE, or SufU, and SufD in addition to SufB and SufC. A number of archaea have SufBCD or SmsCB homologs, whereas a SufS homolog is absent. The mechanisms for sulfur provision in these organisms are unknown, but some S^0^-reducing organisms, such as the members of *Thermoplasma* and *Ignicoccus*, may utilize mechanisms similar to the S^0^-dependent pathway in *T. kodakarensis*.

In addition to the SmsB, SmsC, and CSD homologs, *T. kodakarensis* also harbors a homolog of the Fe-S cluster carrier protein SufT (TK1693). Although the results of *in vitro* analysis indicated that *Tk*-SmsB_2_C_2_ is able to directly transfer the generated Fe-S cluster to LipS (Fig. 5 and 6), further investigation will be required to clarify whether SufT can transfer Fe-S clusters and enhance the transfer efficiency in *T. kodakarensis* cells.

## MATERIALS AND METHODS

### Chemicals, strains, media, and culture conditions

Unless mentioned otherwise, chemical reagents were purchased from Nacalai Tesque (Kyoto, Japan) or Fujifilm Wako Pure Chemicals (Osaka, Japan). *T. kodakarensis* KU216 (Δ*pyrF*) (47) and KPD1 (Δ*pyrF*, Δ*pdaD*) were cultured under anaerobic conditions at 85°C in nutrient-rich medium (ASW-YT-m1-S^0^ or ASW-YT-m1-Pyr). *T. kodakarensis* KPD1 is derived from the KU216 strain, in which the *pdaD* gene (TK0149) was disrupted, and thus displays agmatine auxotrophy (48). ASW-YT-m1-S^0^ medium was composed of 0.8× artificial seawater (ASW) (49), 5.0 g L^−1^ yeast extract, 5.0 g L^−1^ tryptone, 2.0 g L^−1^ elemental sulfur, 20 µM KI, 20 µM H_3_BO_3_, 10 µM NiCl_2_·6H_2_O, and 10 µM sodium tungstate. The composition of ASW-YT-m1-Pyr is the same as ASW-YT-m1-S^0^, with elemental sulfur replaced by sodium pyruvate (5.0 g L^−1^). *E. coli* strain DH5α (Takara Bio, Kusatsu, Japan) used for plasmid construction, and *E. coli* strains BL21-CodonPlus(DE3)-RIL (Agilent Technologies, Santa Clara, CA, USA) and Rosetta-gami 2(DE3) (EMD Millipore, Billerica, MA, USA) used for gene expression were cultivated at 37°C in lysogeny broth (LB) medium containing ampicillin (100 mg liter^−1^). Chloramphenicol (30 mg liter^−1^) was also added to the LB medium to cultivate *E. coli* BL21-CodonPlus(DE3)-RIL or Rosetta-gami 2(DE3) cells.

### Gene expression and purification of TK0730, TK0731, and TK1990 recombinant proteins

Plasmids for the expression of TK1990 and TK0731 genes were constructed as follows. Coding regions of TK1990 and TK0731 with NheI-EcoRI (for TK1990) and NdeI-BamHI (for TK0731) restriction sites were amplified from the genomic DNA of *T. kodakarensis* KU216 by PCR with the primer sets eTK1990-F/-R and eTK0731-F/-R (Table 2), respectively, and inserted into plasmid pET-21a(+) at the corresponding sites. The resulting plasmids, including TK1990 and TK0731, were named pET1990 and pET0731, respectively. After confirming the absence of unintended mutations by DNA sequencing, pET1990 and pET0731 were individually introduced into *E. coli* strain BL21-CodonPlus(DE3)-RIL. Transformants were cultivated at 37^ o^C until OD_660_ reached 0.4–0.6, and gene expression was induced by the addition of isopropyl β-d-1-thiogalactopyranoside (IPTG) at a final concentration of 0.1 mM. Cells were further cultivated at 37^ o^C for 7 h, harvested, resuspended in 50 mM Tris-HCl buffer (pH 7.5), and disrupted by sonication. After centrifugation (4^o^C, 13,000 × *g*, 15 min), the soluble cell-free extracts were incubated at 75^ o^C for 15 min to remove thermolabile proteins from *E. coli*. After centrifugation (4^ o^C, 13,000 × *g*, 20 min), the supernatants including the TK1990 and TK0731 recombinant proteins were individually applied to an anion-exchange column, ResourceQ (GE Healthcare, Little Chalfont, UK), and proteins were eluted with a linear gradient of NaCl (0-1.0 M) in 50 mM Tris-HCl (pH 7.5) at a flow rate of 2.0 mL min^−1^. After exchanging the buffer of the relevant fractions using Amicon Ultra centrifugal filter unit (MWCO 10 K) (EMD Millipore), the proteins were separated by a gel filtration column, Superdex 200 Increase 10/300 Gl (GE Healthcare), with a mobile phase of 50 mM HEPES (pH 7.5) including 0.15 M NaCl at a flow rate of 0.5 mL min^−1^. Relevant fractions were concentrated using an Amicon Ultra centrifugal filter unit (MWCO 10 K).

**TABLE 2:** Primers used in this study

Primer name	Sequence (5' to 3')
eTK1990-F	ATACATATGGCTAGCATGAAGATACCGGACGATATTAGAAAG
eTK1990-R	CGGAGCTCGAATTCTTAACCTCTCAAACCTCTTATCAGATTC
eTK0731-F	GAAGGAGATATACATATGCTCAAAGTTGAGAACCTTCACG
eTK0731-R	GCTCGAATTCGGATCCTCATGCTCCCACCTCCTCGAATATCCTG
eTK0730-nHis-F	GTGTTGTCATATGCACCATCACCATCACCATACTGAAACTATAACAATG
eTK0730-nHis-R	GAATTGCCCAGTCGACTCACATGCCCCCGCTTAC

The TK0730 gene was expressed in either its native host *T. kodakarensis* under the control of the strong promoter *Pcsg*, the promoter for the cell-surface glycoprotein gene TK0895, or *E. coli*. The plasmids for the overexpression of TK0730 were constructed as follows. Coding region of TK0730 with NdeI-SalI restriction sites was amplified by PCR using a primer set eTK0730-nHis-F/-R (Table 2), and a 6xHis tag sequence was incorporated in the N-terminus for purification. The amplified gene fragment was digested with NdeI and SalI enzymes and inserted into (i) a *T. kodakarensis-E. coli* shuttle plasmid previously used for overexpression of TK2101 (50) (named as pRPETK0730) or (ii) plasmid pET-21a(+) (named as pET0730). After confirming the absence of unintended mutations by DNA sequencing, the expression plasmid pRPETK0730 was introduced into the host strain *T. kodakarensis* KPD1 (Δ*pyrF*, Δ*pdaD*) for gene expression. For transformation, *T. kodakarensis* KPD1 was cultivated in ASW-YT-m1-S^0^ medium supplemented with 1.0  mM agmatine at 85°C for 12 h. Cells were harvested, resuspended with 200 µL of 0.8× ASW-m1 (0.8× ASW with 20 µM KI, 20 µM H_3_BO_3_, 10 µM NiCl_2_·6H_2_O, and 10 µM sodium tungstate), and kept on ice for 30 min. The expression plasmid pRPETK0730 (3.0 µg) was added to the cells, and the mixtures were kept on ice for 1 h. After heat shock at 85°C for 45 s, the mixtures were kept on ice for 10 min. Cells were inoculated into ASW-YT-m1-S^0^ liquid medium and incubated at 85°C for 24 h twice. Cells were spread onto solid ASW-YT-m1-S^0^ medium. After incubation at 85°C for 24 h, transformants displaying agmatine prototrophy were isolated, and the presence of recombinant plasmids and absence of unintended mutations were confirmed by PCR and DNA sequencing, respectively. One of the transformants was designated *T. kodakarensis* ETK0730 and utilized for further experiments. The expression plasmid pET0730 was introduced into *E. coli* strain Rosetta-gami 2(DE3).

The ETK0730 strain was cultivated in ASW-YT-m1-Pyr at 85°C for 20 h. Cells were resuspended in 20 mM sodium phosphate buffer (pH 7.4) containing 500 mM NaCl and 20 mM imidazole and disrupted by sonication. After centrifugation (4^ o^C, 13,000 × *g*, 15 min), the supernatant was loaded onto a His GraviTrap column (Cytiva, Marlborough, MA, USA). Proteins were eluted with elution buffer I (pH 7.4) containing 20  mM sodium phosphate, 500  mM NaCl, and 500  mM imidazole. After exchanging the buffer to 50 mM Tris-HCl (pH 7.5), the sample was further purified by anion-exchange chromatography and gel filtration chromatography as described above. Transformants within expression plasmid pET0730 were cultivated at 37^ o^C until OD_660_ reached 0.4–0.6, and gene expression was induced by the addition of IPTG at a final concentration of 0.1 mM. Cells were further cultivated at 25^ o^C for 15 h, harvested, resuspended in 50 mM Tris-HCl buffer (pH 7.5), and disrupted by sonication. The recombinant protein was purified by heat treatment, affinity chromatography, anion-exchange chromatography, and gel filtration chromatography as described above.

Protein concentrations were determined with the Protein Assay System (Bio-Rad, Hercules, CA, USA) using bovine serum albumin (Thermo Fisher Scientific, Waltham, MA, USA) as a standard. The purities of TK0730, TK0731, and TK1990 recombinant proteins were analyzed by SDS-PAGE.

### Protein interaction analysis by gel filtration chromatography

The interaction analysis between TK0730 (*Tk*-SmsB) and TK0731 (*Tk*-SmsC) proteins was performed utilizing gel filtration chromatography with a Superdex 200 Increase 10/300 Gl column. The column was equilibrated with a mobile phase of 50 mM HEPES (pH 7.5) including 0.15 M NaCl at a constant flow rate of 0.5 mL min^−1^. The absorbance derived from protein was monitored at 280 nm. Blue Dextran 2000 was used to determine the void volume, whereas standard proteins (aprotinin [6.5 kDa], ribonuclease A [13.7 kDa], carbonic anhydrase [29 kDa], ovalbumin [43 kDa], conalbumin [75 kDa], and aldolase [158 kDa]) of Gel Filtration Calibration Kits (GE Healthcare) were utilized to prepare a standard curve. *Tk*-SmsB protein (40 µM) alone, *Tk*-SmsC protein (72 µM) alone, or a mixture of *Tk*-SmsB protein (40 µM) and *Tk*-SmsC protein (40 µM) was applied to the gel filtration column. In the examination of the mixture, fractions containing proteins were analyzed by SDS-PAGE. The densities of the protein bands were measured by software ImageJ (51).

For the association analysis of *Tk*-CSD and *Tk*-SmsB/C, *Tk*-CSD protein alone, a mixture of *Tk*-CSD and *Tk*-SmsB, a mixture of *Tk*-CSD and *Tk*-SmsC, and a mixture of *Tk*-CSD, *Tk*-SmsB, and *Tk*-SmsC (30 µM of each protein) were examined. In addition, *Tk*-SmsB protein alone, *Tk*-SmsC protein alone, and a mixture of *Tk*-SmsB and *Tk*-SmsC (30 µM of each protein), as the controls, were also analyzed. The collected fractions were analyzed by SDS-PAGE.

To confirm whether *Tk*-CSD interacts with *Tk*-SmsB, additional affinity chromatography was carried out. A mixture (200 µL) of *Tk*-CSD and *Tk*-SmsB (30 µM of each protein) was loaded onto a His GraviTrap column, and the wash and eluate fractions were collected for analysis (there was no flow-through portion since the loading volume did not exceed that of the column). *Tk*-SmsB protein alone and *Tk*-CSD protein alone were used as the controls. The collected fractions were analyzed by SDS-PAGE.

### Pull-down assay of *Tk*-SmsB

A pull-down experiment was carried out based on the reported method and protocol (52) with some modifications. The purified His-tagged *Tk*-SmsB protein and the cell lysate of the KU216 strain were used as the bait and prey, respectively. *Tk*-SmsB protein (100 µg) (together with or without an equal molar concentration of *Tk*-SmsC) and the cell lysate (10 mg of general amount of protein) were well mixed and incubated at 60°C for 30 min. The resulting mixture (1 mL) was loaded onto a His GraviTrap column. The column was sequentially washed by equilibrium buffer (20 mM Tris-HCl, pH 7.5, 150 mM NaCl), wash buffers I (20 mM Tris-HCl, pH 7.5, 150 mM NaCl, 20 mM imidazole) and II (20 mM Tris-HCl, pH 7.5, 150 mM NaCl, 50 mM imidazole), and elution buffer II (20 mM Tris-HCl, pH 7.5, 150 mM NaCl, 500 mM imidazole). Cell lysate without *Tk*-SmsB was used as a negative control. To further exclude the false positives resulting from nonspecific interactions of cell lysate proteins, an additional negative control containing cell lysate and an irrelevant His-tagged protein TK2101 (49.7 kDa), an aminotransferase of *T. kodakarensis* (50), was also analyzed. The eluate fractions were analyzed by SDS-PAGE.

### ATPase assay

The hydrolysis of ATP by the recombinant *Tk*-SmsC protein was measured in the presence or absence of *Tk*-SmsB protein. The reaction mixture (25 µL) contained 50 mM HEPES (pH 7.5 at 75°C), 5 mM MgCl_2_, 5 mM ATP, and 0.5 µg of *Tk*-SmsC protein with or without 0.9 µg of *Tk*-SmsB protein. The reaction mixtures without *Tk*-SmsC were set as blank controls. After pre-incubation at 75°C for 1 min, the reaction was initiated by adding ATP. After incubation at 75°C for 3, 4, and 5 min, the reaction was stopped by rapid cooling on ice for 5 min and removal of proteins by ultrafiltration using an Amicon Ultra centrifugal filter unit (MWCO 10 K). The ATPase assay was performed in triplicate. The generated ADP was quantified by HPLC.

For the measurement of the kinetic parameters, ATP was varied from 0.05 to 10 mM (0.05, 0.1, 0.2, 0.4, 0.8, 1, 1.5, 2, 2.5, 3, 4, 5, 6, 8, and 10 mM) in the presence of 5 mM MgCl_2_ and 0.2 µg (for 0.05–1.5 mM ATP) or 0.4 µg (for 2–10 mM ATP) of *Tk*-SmsC protein with or without equal molar concentration of *Tk*-SmsB protein. The reaction mixture without protein(s) was set as a blank control. After pre-incubation at 75°C for 1 min, the reaction was initiated by adding ATP. After incubation at 75°C for 3, 4, and 5 min, the reaction was stopped by rapid cooling on ice for 5 min and removal of proteins by ultrafiltration using an Amicon Ultra centrifugal filter unit (MWCO 10 K). The assay was performed in triplicate. The generated ADP was quantified by HPLC. Kinetic parameters were obtained by fitting the data to the Michaelis-Menten equation using IGOR Pro, version 6.03 (Wave-Metrics, Lake Oswego, OR).

### *In vitro* Fe-S cluster assembly on TK0730-TK0731 complex

To remove any possible residual Fe atoms in the purified *Tk*-SmsB protein sample as well as that of the *Tk*-SmsC protein, the apo-forms of these proteins were prepared according to previous methods (30, 53). The purified proteins (0.1 mM of each) were individually incubated with 5 mM ethylenediaminetetraacetic acid (EDTA) and 2 mM potassium ferricyanide (PFC) (molar ratios of 1:50:20) on ice for 30 min, followed by desalting with a PD-10 column (GE Healthcare). Two methods were tested to examine the assembly of Fe-S clusters. All the following operations were performed under anaerobic conditions except for centrifugation. In method (i), chemical reconstitution of the Fe-S cluster was performed as follows. Apo-*Tk*-SmsB (30 µM), together with or without apo-*Tk*-SmsC protein (30 µM), was treated with 10 mM dithiothreitol (DTT) and added to reconstitution buffer I (50 mM HEPES buffer [pH 8.0], 50 mM NaCl, 5 mM DTT, and 10% [vol/vol] glycerol). FeCl_3_ and Na_2_S were slowly added to the mixtures up to final concentrations of 150 µM and 300 µM, respectively. When necessary, 1 mM ATP and 1 mM MgCl_2_ were also added into the mixture. The resulting mixtures (3 mL) were then incubated at room temperature for 1 h. The precipitates were removed by centrifugation (4^ o^C, 13,000 *× g*, 15 min). In order to exclude the effects of free Fe-S clusters not associated with the scaffold proteins, the supernatants were treated with 5 mM EDTA for 10 min to disrupt these labile clusters and chelate the iron cations. After desalting with 50 mM HEPES buffer (pH 8.0) via a PD-10 column, the reconstituted proteins were concentrated with Amicon Ultra filters (MWCO 10K). In method (ii), CSD-based reconstitution of the Fe-S cluster was carried out as follows. Apo-*Tk*-SmsB (20 µM), together with or without apo-*Tk*-SmsC protein (20 µM), was incubated with reconstitution buffer II (50 mM HEPES buffer [pH 8.0 at 60°C], 100 mM NaCl, and 10 mM DTT), 5-fold molar excess of FeCl_3_ (100 µM), 2.5 µM recombinant *Tk*-CSD (TK1990) protein, 2 mM cysteine, 0.5 mM pyridoxal phosphate (PLP), 2 mM MgCl_2_, and 5 mM ATP at 60°C for 30 min. After the treatment with EDTA, the resulting mixtures (400 µL) were desalted and then concentrated, as described in method (i).

The UV-visible spectra of assembled Fe-S clusters on proteins were monitored with a UV-1800 spectrophotometer (SHIMADZU, Kyoto, Japan) at room temperature. CD spectra were recorded using 300 µL of 100 µM holo-*Tk*-SmsB_2_C_2_ complex (chemically reconstituted) or its apo-form by a CD spectrometer J-1500 (JASCO Corporation, Tokyo, Japan). The sulfide and iron content of the chemically reconstituted *Tk*-SmsB_2_C_2_ complex was determined as previously described (54–56). Briefly, sulfide was measured by its conversion to methylene blue in a mixture containing 20 mM *N*,*N*-dimethyl-1,4-phenylenediamine sulfate (in 7.2 M HCl) and 30 mM FeCl_3_ (in 1.2 M HCl) (1:1 in volume). After color development, the absorbance at 650 nm was measured. The sulfide concentration was determined based on a standard Na_2_S curve. Iron quantification was carried out with acid-permanganate, the water-soluble Fe(II) chelator neocuproine, and the spectrophotometric reagent ferrozine. After the formation of the magenta color of the mixture, the absorbance at 562 nm was measured. The ferrous concentration was determined based on a standard FeCl_2_ curve.

### Measurement of Fe-S cluster transfer

The apo-form of *T. kodakarensis* lipoyl synthase LipS (encoded by TK2109 and TK2248) was prepared by incubating with EDTA and PFC as described above. All the following operations were performed under an anaerobic environment except for centrifugation. The apo-LipS protein (30 µM) was incubated with chemically reconstituted holo-*Tk*-SmsB_2_C_2_ complex (30 µM) in reconstitution buffer I at room temperature for 3 h. The resulting mixture (1 mL) was loaded onto a His GraviTrap column, and the flow-through fraction was kept for analysis. Proteins were eluted with elution buffer I. Proteins in the flow-through fraction and eluate were desalted with a PD-10 column with 50 mM HEPES buffer (pH 8.0), concentrated with Amicon Ultra filters (MWCO 10K), and analyzed by SDS-PAGE. The UV-visible spectra of Fe-S clusters on proteins were monitored as described above.

### LipS activation with the *Tk*-SmsB_2_C_2_ complex

LipS reaction coupled with the Fe-S cluster transfer reaction was carried out as follows. All the reactions were carried out under an anaerobic environment. (i) Apo-LipS was incubated with chemically reconstituted holo-*Tk*-SmsB_2_C_2_ complex. The reaction mixture contained 50 mM HEPES buffer (pH 8.0 at 60°C), 10 mM DTT, 2 mM *S*-adenosyl methionine (SAM), 150 µM octanoyl-peptide, 10 mM sodium dithionite, 15 µM apo-LipS1 (TK2109), 15 µM apo-LipS2 (TK2248), and 100 µM holo-*Tk*-SmsB_2_C_2_ complex. The reaction mixtures without apo-LipS1/S2 or holo-*Tk*-SmsB_2_C_2_ complex were used as the controls. The reactions were carried out at 60°C for 30 min. (ii) Apo-*Tk*-SmsB (50 µM), apo-*Tk*-SmsC (50 µM), apo-LipS1 (15 µM), and apo-LipS2 (15 µM) were incubated in a reaction mixture containing reconstitution buffer II, 300 µM FeCl_3_, 2 mM MgCl_2_, 5 mM ATP, together with 2.5 µM *Tk*-CSD, 2 mM cysteine, and 0.5 mM PLP. The reaction mixtures without apo-*Tk*-SmsB_2_C_2_ or apo-LipS were set as the controls. After incubation at 60°C for 15 min, 2 mM SAM, 150 µM octanoyl-peptide, and 10 mM sodium dithionite were added to the reaction mixture. The resulting mixtures were incubated at 60°C for a further 30 min. All of the reactions were stopped by rapid cooling on ice for 5 min and removal of the enzyme by ultrafiltration using Amicon Ultra centrifugal filter units (MWCO 10 K). The reaction products were analyzed by HPLC. The reduced form of standard lipoyl-peptide was prepared by treating lipoyl-peptide with DTT, whereas the intermediate thiol-octanoyl-peptide was prepared by using chemically reconstituted LipS2 protein. Products generated by chemically reconstituted holo-LipS1/S2 were back to 15% solvent B and also analyzed as a positive control.

### Measurement and identification of reaction products by HPLC

The HPLC analysis was carried out with a COSMOSIL 5C_18_-PAQ column (4.6 mm × 250 mm, 5 µm particle size). The ADP produced by *Tk*-SmsC was quantified as follows. The mobile phase consisted of solvent A (20 mM potassium phosphate buffer with 5 mM tetrabutylammonium hydrogen sulfate, pH 6.0) and solvent B (acetonitrile). A gradient of 0%–45% solvent B was applied from 0 to 15 min, followed by a 1 min maintenance; then, the proportion was returned to 15% solvent B, and the column was re-equilibrated for 4 min. A flow rate of 0.7 mL min^−1^ was maintained throughout the procedure. Absorbance at 259 nm was monitored by a UV detector SPD-M20A (SHIMADZU).

The reaction products of LipS were analyzed as follows. The mobile phase consisted of solvent C (MilliQ water with 0.1% formic acid) and solvent D (acetonitrile with 0.1% formic acid). A gradient of 15-20% solvent D was applied from 0 to 5 min, followed by a second gradient of 20%–30% solvent D from 5 to 30 min, and a third gradient of 30%–40% solvent D from 30 to 35 min; then, the proportion returned back to 15% solvent D, and the column was re-equilibrated for 15 min. A flow rate of 0.7 mL min^−1^ was maintained throughout the procedure. Absorbance at 200 nm was monitored by a UV detector.

### Sequence alignment and structure modeling

Amino acid sequence alignments were carried out by ClusterX (57) and colored by Web server ESPript 3.0 (58) (http://espript.ibcp.fr/ESPript/ESPript/). The 3D structure model of *Tk*-SmsB was predicted by AlphaFold2 (59). The generated model was validated by the PROCHECK module of UCLA-DOE LAB-SAVES v6.0 (60) (https://saves.mbi.ucla.edu/). The superimposition diagrams of the 3D structures were generated with PyMOL version 2.6 (61).

### Bioinformatic analysis

Gene distribution analysis was performed using the BLAST search tool in the KEGG database (62, 63) (https://www.genome.jp/tools/blast/). The amino acid sequence of *Tk*-CSD (TK1990) was used as the query to search for the corresponding homologs in archaea. The threshold of the E-values was 6e–12 for CSD homologs.

The phylogenetic trees were constructed in MEGA12 (64) by using the Maximum Likelihood method and the LG model (65) with the default parameters.

## Data Availability

NCBI accession numbers for TK0730, TK0731, and TK1990 are BAD84919, BAD84920, and BAD86179, respectively. All relevant data are included in the article and supplemental material.

## SUPPLEMENTAL MATERIAL

Supplemental material aem.01438-25-s0001.pdf Table S1; Fig. S1 to S8.

ASM does not own the copyrights to Supplemental Material that may be linked to, or accessed through, an article. The authors have granted ASM a non-exclusive, world-wide license to publish the Supplemental Material files. Please contact the corresponding author directly for reuse.
