# Supplementary material for: SmsB-SmsC machinery in Thermococcus kodakarensis functions as an archaeal scaffold mediating Fe-S cluster assembly
Source: Appl Environ Microbiol. 2025 Aug 18;91(9):e01438-25. doi: 10.1128/aem.01438-25 (PMC12442378; doi:10.1128/aem.01438-25)
Supplement: Supplemental material — Table S1; Fig. S1 to S8. [file aem.01438-25-s0001.pdf]

Supplemental material

SmsB-SmsC machinery in *Thermococcus kodakarensis* functions as an archaeal scaffold mediating Fe-S cluster assembly

Jian-qiang Jin[a], Takaaki Sato[ab], and Haruyuki Atomi[ab]

[a] Department of Synthetic Chemistry and Biological Chemistry, Graduate School of Engineering, Kyoto University, Kyoto, Japan
[b] Integrated Research Center for Carbon Negative Science, Kyoto University, Kyoto, Japan.

Corresponding author: Haruyuki Atomi (atomi.haruyuki.8r@kyoto-u.ac.jp)

This file includes
Supplementary Table S1
Supplementary Figures S1 to S8

Table S1. Organism codes and distribution of CSD homologs in selected species of *Archaea*

Order	Organism code	Organism	CSD homolog
Archaeoglobales	afu	*Archaeoglobus fulgidus* DSM 4304	AF_0186<br>AF_0564
	fpl	*Ferroglobus placidus*	Ferp_1122
Halobacteriales	hmu	*Halomicrobium mukohataei* DSM 12286	**Hmuk_1242**
	nph	*Natronomonas pharaonis*	**NP_0936A**<br>(NP_0790A)
	hje	*Halalkalicoccus jeotgali* B3	**HacjB3_01375**<br>(HacjB3_08320)
Natrialbales	nge	*Natronobacterium gregoryi* SP2	**Natgr_1522**
Methanobacteriales	mcub	*Methanobacterium congolense* isolate Buetzberg	**MCBB_2234**<br>(MCBB_1046)
	mru	*Methanobrevibacter ruminantium* M1	mru_1568
	mst	*Methanosphaera stadtmanae* DSM 3091	Msp_1002
	mth	*Methanothermobacter thermautotrophicus* Delta H	**MTH_1389**
Methanococcales	mfe	*Methanocaldococcus fervens* AG86	
	mja	*Methanocaldococcus jannaschii* DSM 2661	
	mmp	*Methanococcus maripaludis* S2	
Methanocellales	mez	*Methanocella conradii* HZ254	**Mtc_1262**<br>(Mtc_1595)
Methanomicrobiales	mpi	*Methanolacinia petrolearia* DSM 11571	Mpet_2792<br>Mpet_2673
	mbn	*Methanoregula boonei* 6A8	**Mboo_1984**<br>**Mboo_2054**<br>(Mboo_0356<br>Mboo_2125<br>Mboo_0298)
	mhu	*Methanospirillum hungatei* JF-1	**Mhun_2219**<br>**Mhun_2203**<br>(Mhun_2209<br>Mhun_0255<br>Mhun_2213)
Methanosarcinales	mbu	*Methanococcoides burtonii* DSM 6242	**Mbur_2068**<br>(Mbur_0289)
Methanosarcinales	mpy	*Methanolobus psychrophilus* R15	**Mpsy_1021**<br>**Mpsy_2236**<br>**Mpsy_2948**<br>(Mpsy_2007<br>Mpsy_0210)
	mac	*Methanosarcina acetivorans* C2A	**MA_0236**<br>(MA_2718<br>MA_3264<br>MA_0808<br>MA_1950)
	mma	*Methanosarcina mazei* Go1	**MM_1517**<br>(MM_0109<br>MM_1955)
Thermococcales	ppac	*Palaeococcus pacificus* DY20341	**PAP_01245**<br>(PAP_08965<br>PAP_01530)
	pab	*Pyrococcus abyssi* GE5	**PAB0157**<br>(PAB0943)
	pfu	*Pyrococcus furiosus* DSM 3638	**PF0164**<br>(PF1066)
	pho	*Pyrococcus horikoshii* OT3	PH0626
	pya	*Pyrococcus yayanosii* CH1	**PYCH_19110**
	tba	*Thermococcus barophilus* MP	**TERMP_01439**<br>(TERMP_00841)
	tch	*Thermococcus chitonophagus* 1	**CHITON_0384**<br>(CHITON_0890)
	teu	*Thermococcus eurythermalis* A501	**TEU_06070**
	tga	*Thermococcus gammatolerans* EJ3	**TGAM_1311**
	tic	*Thermococcus indicus* IOH1	**FH039_03850**
	tko	*Thermococcus kodakarensis* KOD1	**TK1990**
	tlt	*Thermococcus litoralis* DSM 5473	**OCC_11247**
	ton	*Thermococcus onnurineus* NA1	**TON_0289**
	tsi	*Thermococcus sibiricus* MM 739	**TSIB_0193**
Thermoplasmatales	cdiv	*Cuniculiplasma divulgatum* PM4	**CPM_0643**<br>(CPM_1458)
	fai	*Ferroplasma acidiphilum* Y	FAD_0633
	tac	*Thermoplasma acidophilum* DSM 1728	
	abi	*Aciduliprofundum boonei* T469	
Desulfurococcales	ape	*Aeropyrum pernix* K1	**APE_2023**<br>(APE_1754.1)
	dmu	*Desulfurococcus mucosus* DSM 2162	**Desmu_0940**
	iho	*Ignicoccus hospitalis* KIN4/I	
	iis	*Ignicoccus islandicus* DSM 13165	
	iag	*Ignisphaera aggregans* DSM 17230	**Igag_1781**<br>(Igag_1351)
	smr	*Staphylothermus marinus* F1	**Smar_0379**<br>**Smar_0683**
	tag	*Thermosphaera aggregans* DSM 11486	**Tagg_0269**
	hbu	*Hyperthermus butylicus* DSM 5456	
	pfm	*Pyrolobus fumarii* 1A	**Pyrfu_0712**
Sulfolobales	abri	*Acidianus brierleyi* DSM 1651	DFR85_01430
	aho	*Acidianus hospitalis* W1	Ahos_2325
	mcn	*Metallosphaera cuprina* Ar-4	Mcup_1360
	mse	*Metallosphaera sedula* DSM 5348	Msed_0737
	sso	*Saccharolobus solfataricus* P2	SSO2215
	sai	*Sulfolobus acidocaldarius* DSM 639	Saci_0024
	sih	*Sulfolobus islandicus* HVE10/4	SiH_0032
	sto	*Sulfurisphaera tokodaii* 7	STK_21400
Thermofilales	tpe	*Thermofilum pendens* Hrk 5	**Tpen_0949**
Thermoproteales	cma	*Caldivirga maquilingensis* IC-167	
	pai	*Pyrobaculum aerophilum* IM2	
	pcl	*Pyrobaculum calidifontis* JCM 11548	
	ttn	*Thermoproteus tenax* Kra 1	

**E-values ≤ 3e-62**
1e-55 ≤ E-values ≤ 6e-12

[a] The sequence of the TK1990 protein was used as the query. CSD homologs whose E-values were lower than or equal to 3e-62 are shown with white bold letters on a black background, while those whose E-values were between 1e-55 and 6e-12 are shown with black letters on a gray background. The locus tag shown with white letters in the parentheses on black background indicate second CSD homologs with E-values between 1e-55 and 6e-12.

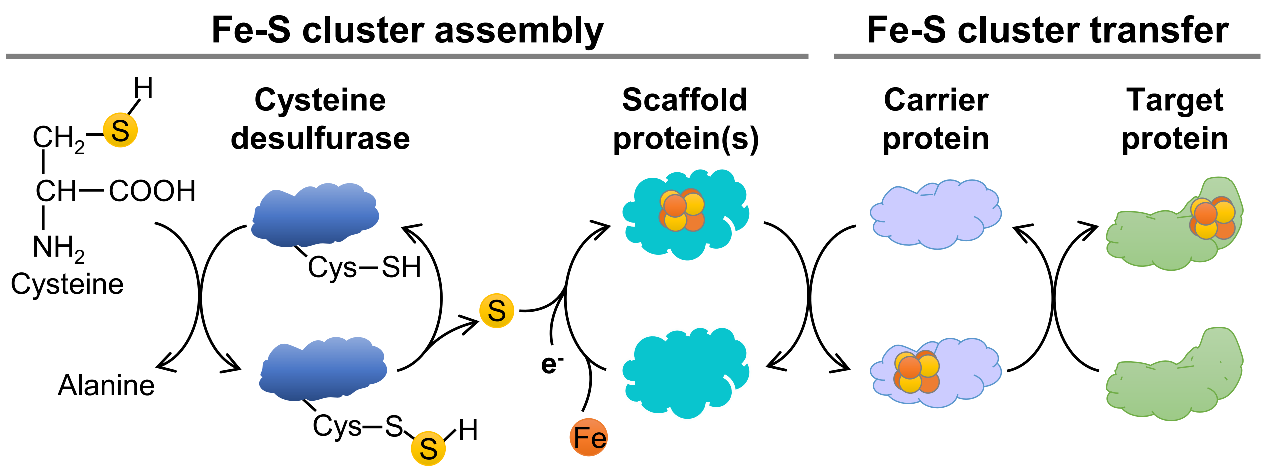

Fig. S1. General scheme of Fe-S cluster biogenesis. Cysteine is desulfurized by cysteine desulfurase, and the released sulfur is transiently bound to the enzyme in the form of a persulfide. The sulfur atom is then transferred to the scaffold protein to generate Fe-S cluster. Finally, the newly assembled Fe-S cluster is transferred to a target apoprotein directly or via carrier protein(s).

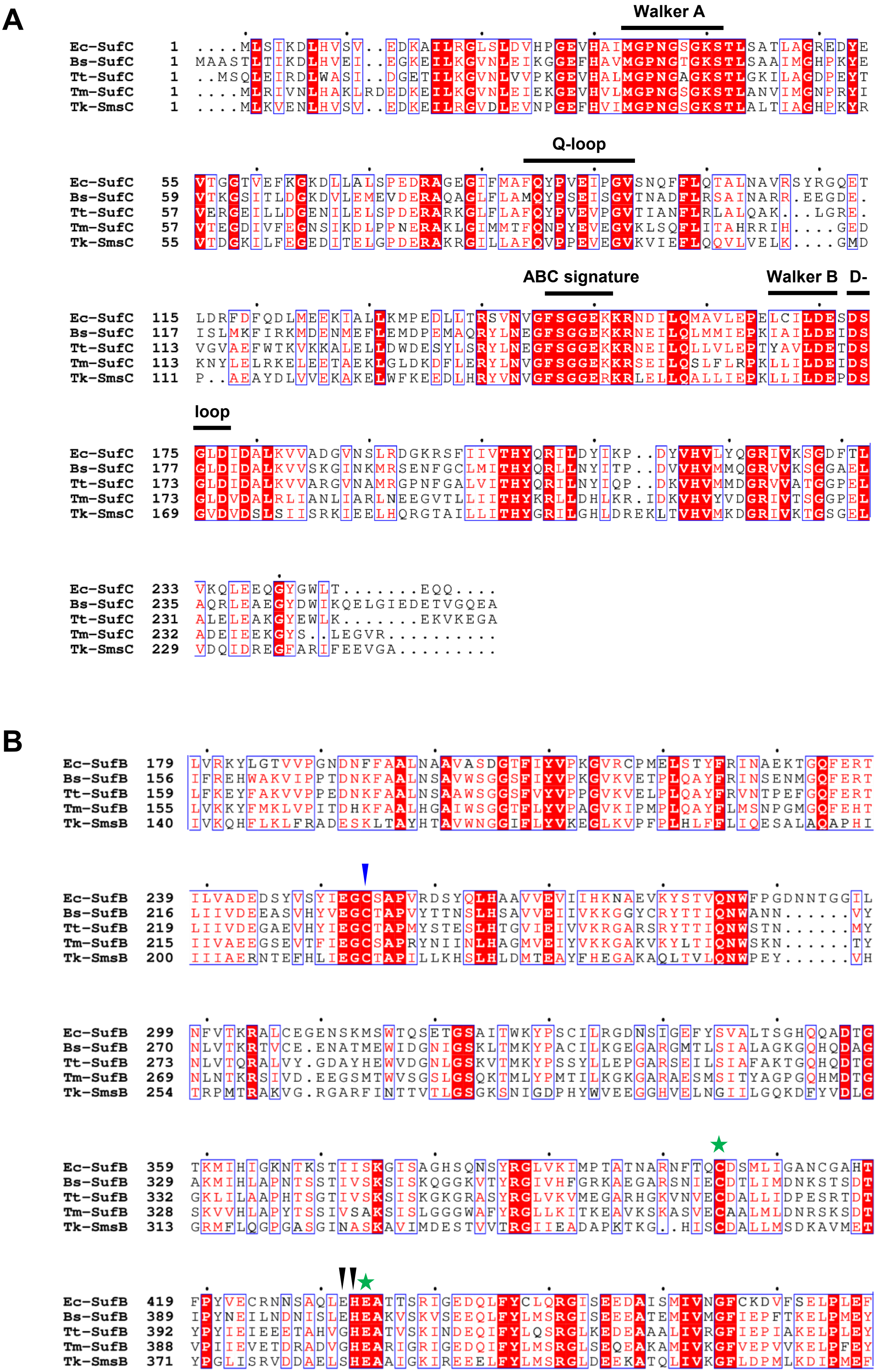

A
Walker A
Ec-SufC 1 ....MLSIKDLHVSV..EDKAILRGLSLDVHPGEVHAIMGPNGSGKSTLSATLAGREDYE
Bs-SufC 1 MAASTLTIKDLHVEI..EGKEILKGVNLEIKGGEFHAVMGPNGTGKSTLSAAIMGHPKYE
Tt-SufC 1 ..MSQLEIRDLWASI..DGETILKGVNLVVPKGEVHALMGPNGAGKSTLGKILAGDPEYT
Tm-SufC 1 ....MLRIVNLHAKLRDEDKEILKGVNLEIEKGEVHVLMGPNGSGKSTLANVIMGNPRYI
Tk-SmsC 1 ....MLKVENLHVSV..EDKEILRGVDLEVNPGEFHVIMGPNGSGKSTLALTIAGHPKYR
Q-loop
Ec-SufC 55 VTGGTVEFKGKDLLALSPEDRAGEGIFMAFQYPVEIPGVSNQFFLQTALNAVRSYRGQET
Bs-SufC 59 VTKGSITLDGKDVLEMEVDERAQAGLFLAMQYPSEISGVTNADFLRSAINARR.EEGDE.
Tt-SufC 57 VERGEILLDGENILELSPDERARKGLFLAFQYPVEVPGVTIANFLRLALQAK...LGRE.
Tm-SufC 57 VTEGDIVFEGNSIKDLPPNERAKLGIMMTFQNPYEVEGVKLSQFLITAHRRIH....GED
Tk-SmsC 55 VTDGKILFEGEDITELGPDERAKRGILLAFQVPPEVEGVKVIEFLQQVLVELK....GMD
ABC signature
Walker B
D-
Ec-SufC 115 LDRFDFQDLMEEKIALLKMPEDLLTRSVNVGFSGGEKKRNDILQMAVLEPELCILDESDS
Bs-SufC 117 ISLMKFIRKMDENMEFLEMDPEMAQRYLNEGFSGGEKKRNEILQLMMIEPKIAILDEIDS
Tt-SufC 113 VGVAEFWTKVKKALELLDWDESYLSRYLNEGFSGGEKKRNEILQLLVLEPTYAVLDETDS
Tm-SufC 113 KNYLELRKELEETAEKLGLDKDFLERYLNVGFSGGEKKRSEILQSLFLRPKLLILDEIDS
Tk-SmsC 111 P..AEAYDLVVEKAKELWFKEEDLHRYVNVGFSGGERKRLELLQALLIEPKLLILDEPDS
loop
Ec-SufC 175 GLDIDALKVVADGVNSLRDGKRSFIIVTHYQRILDYIKP..DYVHVLYQGRIVKSGDFTL
Bs-SufC 177 GLDIDALKVVSKGINKMRSENFGCLMITHYQRLLNYITP..DVVHVMMQGRVVKSGGAEL
Tt-SufC 173 GLDIDALKVVARGVNAMRGPNFGALVITHYQRILNYIQP..DKVHVMMDGRVVATGGPEL
Tm-SufC 173 GLDVDALRLIANLIARLNEEGVTLLIITHYKRLLDHLKR.IDKVHVYVDGRIVTSGGPEL
Tk-SmsC 169 GVDVDSLSIISRKIEELHQRGTAILLITHYGRILGHLDREKLTVHVMKDGRIVKTGSGEL
Ec-SufC 233 VKQLEEQGYGWLT.......EQQ....
Bs-SufC 235 AQRLEAEGYDWIKQELGIEDETVGQEA
Tt-SufC 231 ALELEAKGYEWLK.......EKVKEGA
Tm-SufC 232 ADEIEEKGYS..LEGVR..........
Tk-SmsC 229 VDQIDREGFARIFEEVGA.........
B
Ec-SufB 179 LVRKYLGTVVPGNDNFFAALNAAVASDGTFIYVPKGVRCPMELSTYFRINAEKTGQFERT
Bs-SufB 156 IFREHWAKVIPPTDNKFAALNSAVWSGGSFIYVPKGVKVETPLQAYFRINSENMGQFERT
Tt-SufB 159 LFKEYFAKVVPPEDNKFAALNSAAWSGGSFVYVPPGVKVELPLQAYFRVNTPEFGQFERT
Tm-SufB 155 LVKKYFMKLVPITDHKFAALHGAIWSGGTFLYVPAGVKIPMPLQAYFLMSNPGMGQFEHT
Tk-SmsB 140 IVKQHFLKLFRADESKLTAYHTAVWNGGIFLYVKEGLKVPFPLHLFFLIQESALAQAPHI
Ec-SufB 239 ILVADEDSYVSYIEGCSAPVRDSYQLHAAVVEVIIHKNAEVKYSTVQNWFPGDNNTGGIL
Bs-SufB 216 LIIVDEEASVHYVEGCTAPVYTTNSLHSAVVEIIVKKGGYCRYTTIQNWANN......VY
Tt-SufB 219 LIIVDEGAEVHYIEGCTAPMYSTESLHTGVIEIVVKRGARSRYTTIQNWSTN......MY
Tm-SufB 215 IIVAEEGSEVTFIEGCSAPRYNIINLHAGMVEIYVKKGAKVKYLTIQNWSKN......TY
Tk-SmsB 200 IIIAERNTEFHLIEGCTAPILLKHSLHLDMTEAYFHEGAKAQLTVLQNWPEY......VH
Ec-SufB 299 NFVTKRALCEGENSKMSWTQSETGSAITWKYPSCILRGDNSIGEFYSVALTSGHQQADTG
Bs-SufB 270 NLVTKRTVCE.ENATMEWIDGNIGSKLTMKYPACILKGEGARGMTLSIALAGKGQHQDAG
Tt-SufB 273 NLVTQRALVY.GDAYHEWVDGNLGSKVTMKYPSSYLLEPGARSEILSIAFAKTGQHQDTG
Tm-SufB 269 NLNTKRSIVD.EEGSMTWVSGSLGSQKTMLYPMTILKGKGARAESMSITYAGPGQHMDTG
Tk-SmsB 254 TRPMTRAKVG.RGARFINTTVTLGSGKSNIGDPHYWVEEGGHVELNGIILGQKDFYVDLG
Ec-SufB 359 TKMIHIGKNTKSTIISKGISAGHSQNSYRGLVKIMPTATNARNFTQCDSMLIGANCGAHT
Bs-SufB 329 AKMIHLAPNTSSTIVSKSISKQGGKVTYRGIVHFGRKAEGARSNIECDTLIMDNKSTSDT
Tt-SufB 332 GKLILAAPHTSGTIVSKSISKGKGRASYRGLVKVMEGARHGKVNVECDALLIDPESRTDT
Tm-SufB 328 SKVVHLAPYTSSIVSAKSISLGGGWAFYRGLLKITKEAVKSKASVECAALMLDNRSKSDT
Tk-SmsB 313 GRMFLQGPGASGINASKAVIMDESTVVTRGIIEADAPKTKG..HISCDALLMSDKAVMET
Ec-SufB 419 FPYVECRNNSAQLEHEATTSRIGEDQLFYCLQRGISEEDAISMIVNGFCKDVFSELPLEF
Bs-SufB 389 IPYNEILNDNISLEHEAKVSKVSEEQLFYLMSRGISEEEATEMIVMGFIEPFTKELPMEY
Tt-SufB 392 YPYIEIEEETAHVGHEATVSKINDEQIFYLQSRGLKEDEAAALIVRGFIEPIAKELPLEY
Tm-SufB 388 VPIIEVETDRADVGHEARIGRIGEDQIFYLMSRGLSEQEAKAMIVKGFVEPVVKELPFEY
Tk-SmsB 371 YPGLISRVDDAELSHEAAIGKIREEELFYLMSRGLDEEKATQLIVKGFLDPMLKDIPMEF

continued

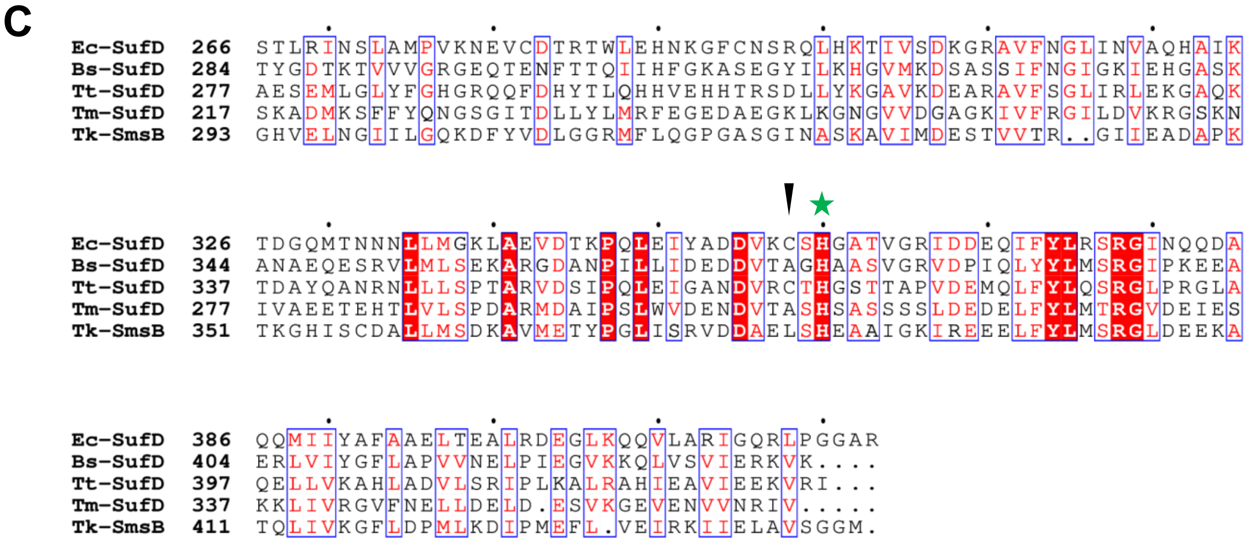

Fig. S2. Amino acid sequence alignment of Suf proteins. (A) Amino acid sequence alignment of the TK0731 protein (*Tk*-SmsC) and SufC proteins from *E. coli* (*Ec*-SufC), *B. subtilis* (*Bs*-SufC), *T. thermophilus* (*Tt*-SufC), and *T. maritima* (*Tm*-SufC). (B) Amino acid sequence alignment of the TK0730 protein (*Tk*-SmsB) and SufB proteins from *E. coli* (*Ec*-SufB), *B. subtilis* (*Bs*-SufB), *T. thermophilus* (*Tt*-SufB), and *T. maritima* (*Tm*-SufB). The residues essential for sulfur-transfer (C254) and coordination of the Fe-S cluster (C405 and E434) in *Ec*-SufB are indicated by a blue arrowhead and green stars, respectively. Two residues (E432 and H433) predicted to be the candidate for the fourth ligand are indicated by black arrowheads. (C) Amino acid sequence alignment of TK0730 (*Tk*-SmsB) and SufD proteins from *E. coli* (*Ec*-SufD), *B. subtilis* (*Bs*-SufD), *T. thermophilus* (*Tt*-SufD), and *T. maritima* (*Tm*-SufD). The critical residue (H360) essential for coordination of the Fe-S cluster in *Ec*-SufD is indicated by a green star. The residue (C358) predicted to be the candidate for the fourth ligand is indicated by a black arrowhead. The sequences (in B and C) are only shown in part. Similar residues in the columns are shown by red letters, and identical residues are shown by white letters on a red background. The columns that display high similarity (global score higher than 0.7) are framed in blue. The dots above the alignment indicate every 10th residue of the top sequence (counting from its initial N-terminal amino acid).

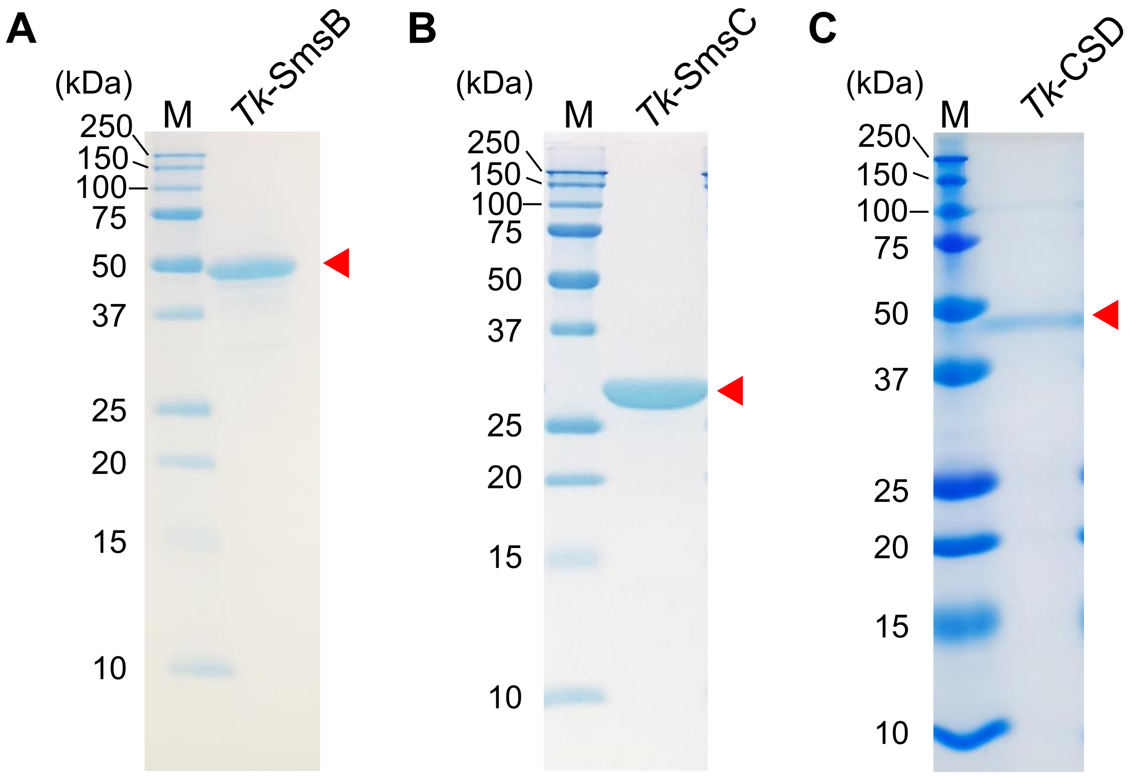

Fig. S3. SDS-PAGE analysis of the purified proteins. Homogeneities of *Tk*-SmsB (A), *Tk*-SmsC (B), and *Tk*-CSD (C) recombinant proteins were analyzed by SDS-PAGE. Three (*Tk*-SmsB) or four (*Tk*-SmsC and *Tk*-CSD) micrograms of purified proteins were applied. The bands of the target proteins are indicated by red arrowheads. Gels were stained with Coomassie Brilliant Blue. M indicates molecular mass marker.

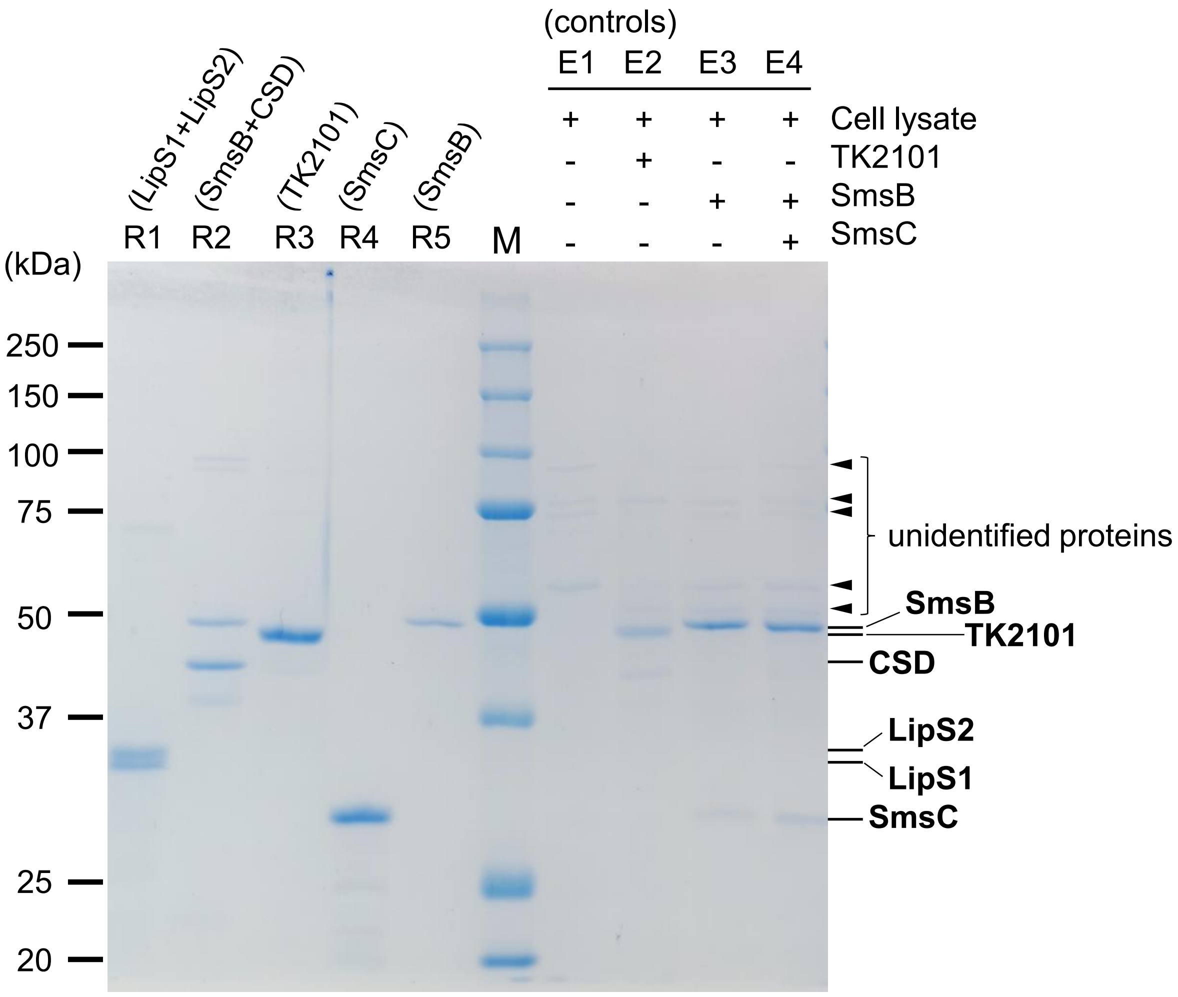

Fig. S4. Pull-down assay of *Tk*-SmsB. Cell lysate of KU216 strain (10 mg of total proteins) was incubated with *Tk*-SmsB (100 μg) in the absence (Lane E3) or presence (Lane E4) of *Tk*-SmsC (equal molar concentration of *Tk*-SmsB). To exclude the false positive signals resulting from nonspecific interactions, the negative controls containing only cell lysate (Lane E1) or cell lysate with an irrelevant His-tagged protein TK2101 (49.7 kDa) (Lane E2) were also examined. After incubation at 60°C for 30 min, the protein mixtures were individually loaded onto a His GraviTrap column. The fractions eluted by 500 mM imidazole were analyzed by SDS-PAGE. Lanes R1-R5 are reference samples. Lane R1, protein mixture of LipS1 (31.7 kDa) and LipS2 (35.2 kDa) (0.5 μg each) (for confirmation of the minimal resolution of the gel); Lane R2, protein mixture of *Tk*-SmsB (50.8 kDa) and *Tk*-CSD (44.4 kDa) (0.5 μg each); lane R3, TK2101 protein (1 μg); Lane R4, *Tk*-SmsC protein (1 μg); lane R5, *Tk*-SmsB protein (0.5 μg). The positions of co-eluted unknown proteins are indicated by black triangles. Gel was stained with Coomassie Brilliant Blue.

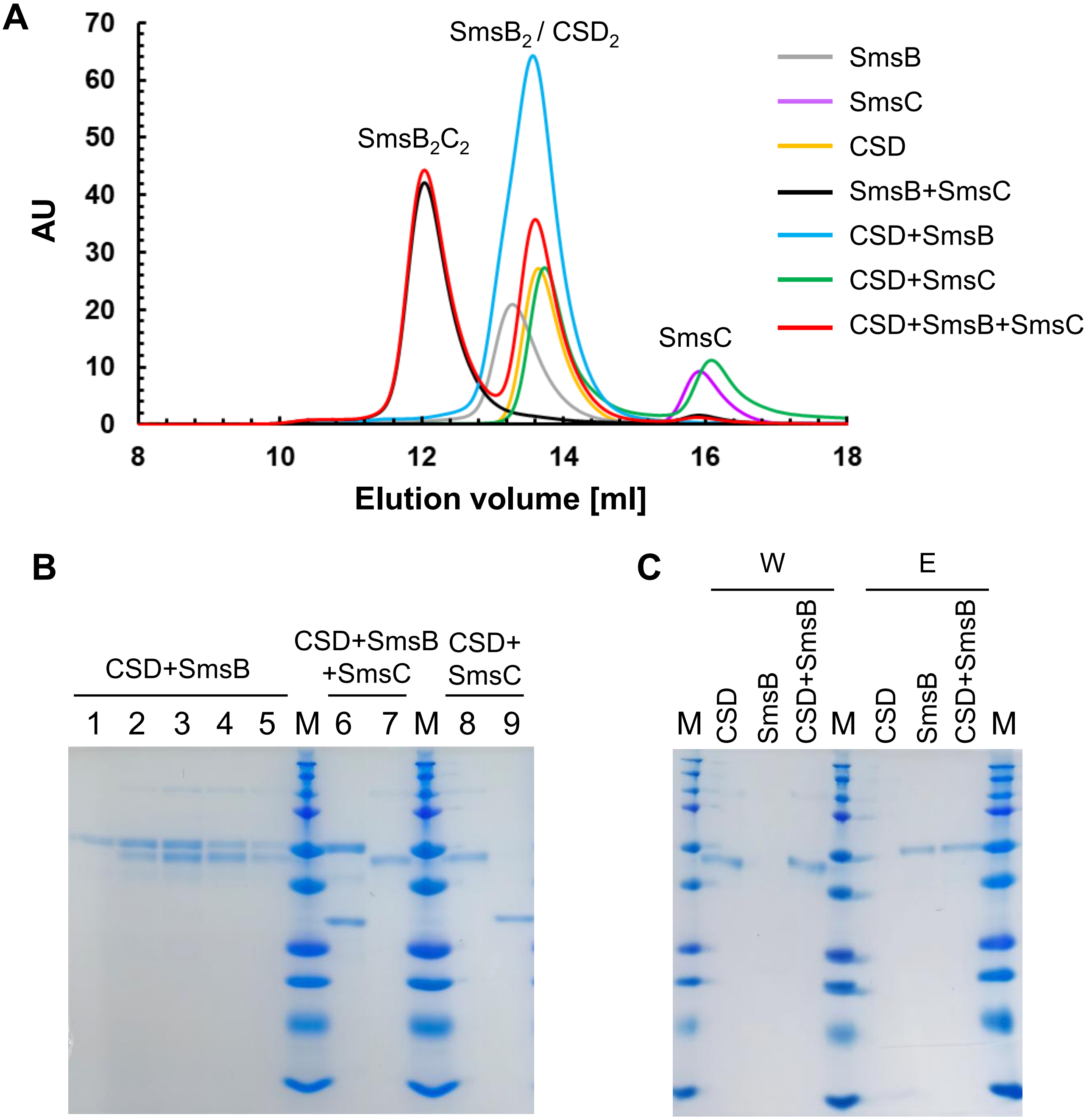

Fig. S5. Association analysis of *Tk*-CSD with *Tk*-SmsB and/or *Tk*-SmsC proteins. (A) Chromatograms of gel filtration chromatography analyzing *Tk*-CSD, *Tk*-SmsB, *Tk*-SmsC, and their mixtures (30 μM of each protein). A Superdex 200 Increase 10/300 GL column was used. (B) SDS-PAGE analysis of the fractions from the gel filtration chromatography. Lanes 1-5, fractions (12.5 to 12.9 ml, 12.9 to 13.3 ml, 13.3 to 13.7 ml, 13.7 to 14.1 ml, and 14.1 to 14.5 ml, respectively) corresponding to the peak of the protein mixture containing *Tk*-CSD and *Tk*-SmsB (blue line in A); Lanes 6 and 7, fractions (11.7 to 12.1 ml, and 13.3 to 13.7 ml, respectively) corresponding to the peaks of the protein mixture containing *Tk*-CSD, *Tk*-SmsB, and *Tk*-SmsC (red line in A); Lanes 8 and 9, fractions (13.3 to 13.7 ml, and 15.7 to 16.1 ml, respectively) corresponding to the peaks of the protein mixture containing *Tk*-CSD and *Tk*-SmsC (green line in A). (C) SDS-PAGE analysis of the fractions from the affinity chromatography. The washing (W) and eluate (E) fractions of the protein solutions, which contained only *Tk*-CSD, only *Tk*-SmsB, and the mixture of *Tk*-CSD and *Tk*-SmsB, were analyzed.

A

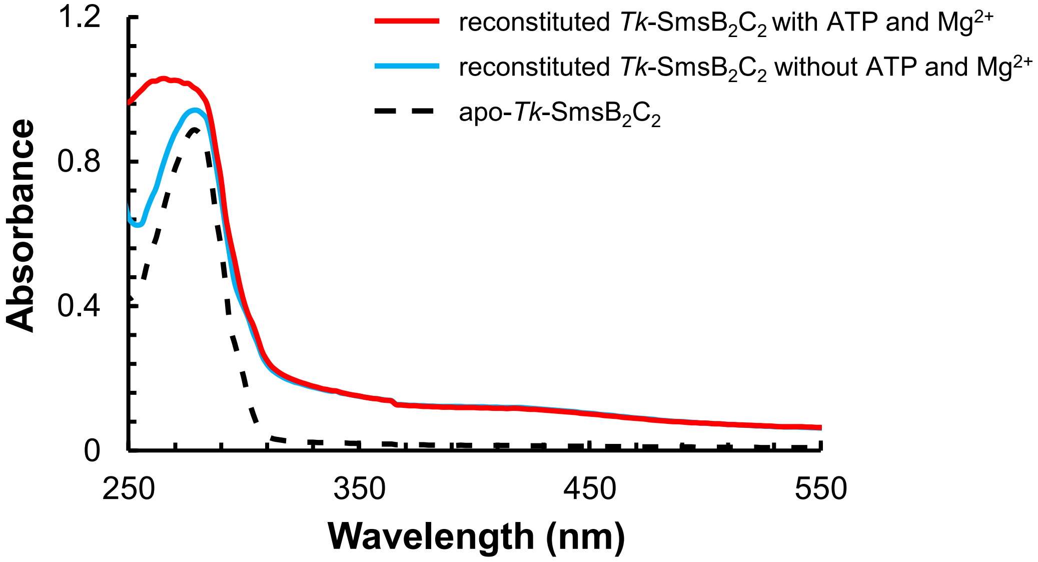

B

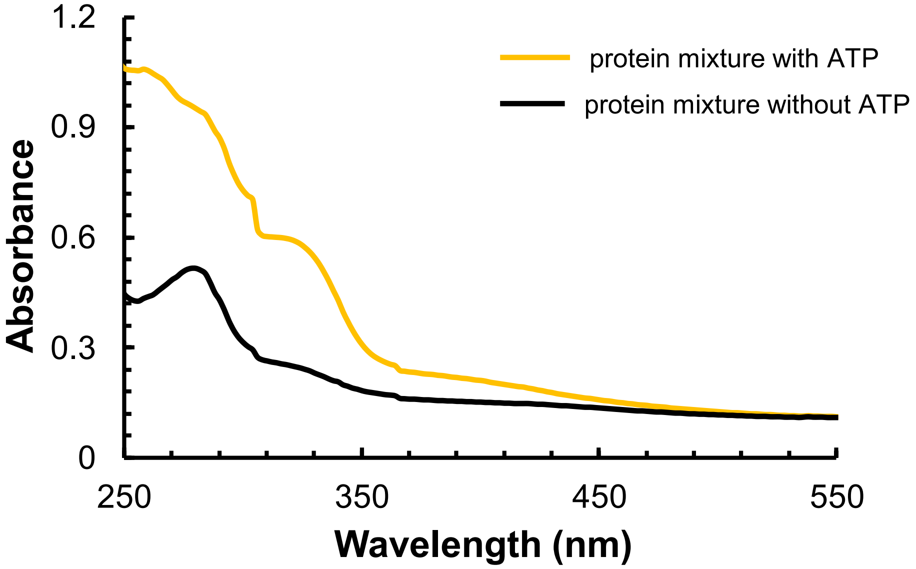

Fig. S6. UV-visible spectra of Fe-S clusters on *Tk*-SmsB$_2$C$_2$ complex. (A) Fe-S clusters on *Tk*-SmsB$_2$C$_2$ complex after chemical reconstitution with or without ATP. The *Tk*-SmsB$_2$C$_2$ complexes (10 μM), before and after chemical reconstitution, were monitored. Black dashed line, *Tk*-SmsB$_2$C$_2$ complex before reconstitution; blue line, *Tk*-SmsB$_2$C$_2$ complex after reconstitution in the absence of ATP and Mg^{2+}; red line, *Tk*-SmsB$_2$C$_2$ complex after reconstitution in the presence of ATP and Mg^{2+}. (B) Fe-S clusters on *Tk*-SmsB$_2$C$_2$ complex via CSD-based assembly. Black line, protein mixture (*Tk*-SmsB, *Tk*-SmsC, and *Tk*-CSD) without ATP (but with Mg^{2+}) after incubation; yellow line, protein mixture with all components (including ATP) after incubation.

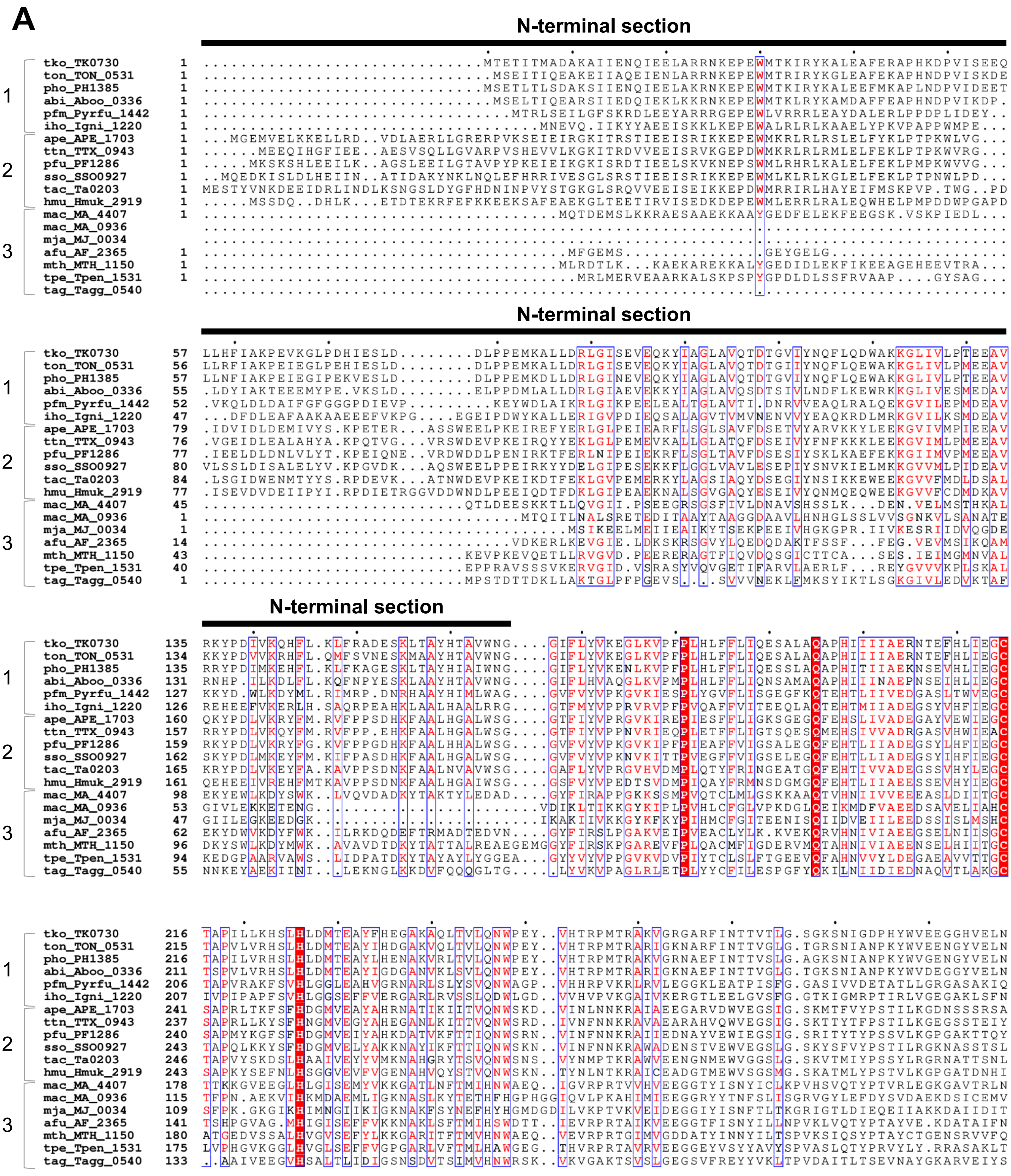
A
N-terminal section
1
2
3
tko_TK0730
ton_TON_0531
pho_PH1385
abi_Aboo_0336
pfm_Pyrfu_1442
iho_Igni_1220
ape_APE_1703
ttn_TTX_0943
pfu_PF1286
sso_SSO0927
tac_Ta0203
hmu_Hmuk_2919
mac_MA_4407
mac_MA_0936
mja_MJ_0034
afu_AF_2365
mth_MTH_1150
tpe_Tpen_1531
tag_Tagg_0540

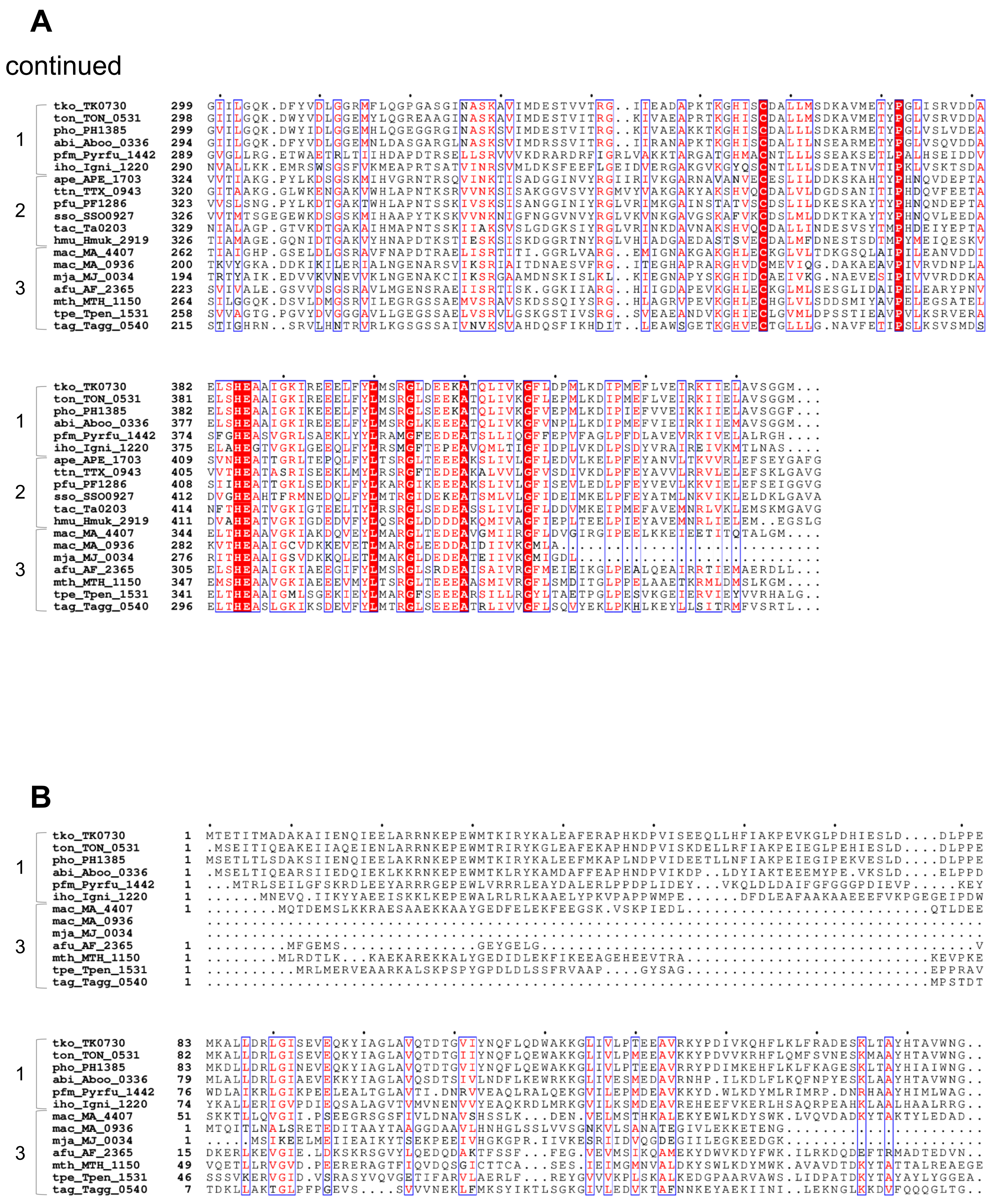
A
continued
1
tko_TK0730 299 GIILGQK.DFYVDLGGRMFLQGPGASGINASKAVIMDESTVVTRG..IIEADAPKTKGHISCDALLMSDKAVMETYPGLISRVDDA
ton_TON_0531 298 GIILGQK.DWYVDLGGEMYLQGREAAGINASKAVIMDESTVITRG..KIVAEAPRTKGHISCDALLMSDKAVMETYPGLVSRVDDA
pho_PH1385 299 GVILGQK.DWYIDLGGEMHLQGEGGRGINASKSVIMDESTVITRG..KIVAEAKKTKGHISCDALLLSDKARMETYPGLVSLVDEA
abi_Aboo_0336 294 GIILGQK.DFYVDLGGEMNLDASGARGLNASKSVIMDRSSVITRG..IIRANAPKTKGHISCDALILNSEARMETYPGLVSQVDDA
pfm_Pyrfu_1442 289 GVGLLRG.ETWAETRLTIIHDAPDTRSELLSRVVVKDRARDRFIGRLVAKKTARGATGHMACNTLLLSSEAKSETLPALHSEIDDV
iho_Igni_1220 290 NVALLKK.EMRSWSGSFVKMEAPRTSATVINRSVMLDKSFEEFLGEIDVERGAKGVKGYQSCNTLLLSDEATNVTIPKLVSKTSDA
2
ape_APE_1703 324 VVTIAKG.PYLKDSGSKMIHVGRNTRSQVINKTISADGGINVYRGIIRIVKGARNAVANVECESLILDDKSKAHTYPHNQVDEPTA
ttn_TTX_0943 320 GITAAKG.GLWKENGAKVWHLAPNTKSRVVNKSISAKGGVSVYRGMVYVAKGAKYAKSHVQCDALVLDGDSANITIPHDQVFEETA
pfu_PF1286 323 VVSLSNG.PYLKDTGAKTWHLAPNTSSKIVSKSISANGGINIYRGLVRIMKGAINSTATVSCDSLILDKESKAYTYPHNQNDEPTA
sso_SSO0927 326 VVTMTSGEGEWKDSGSKMIHAAPYTKSKVVNKNIGFNGGVNVYRGLVKVNKGAVGSKAFVKCDSLMLDDKTKAYTYPHNQVLEEDA
tac_Ta0203 329 NIALAGP.GTVKDTGAKAIHMAPNTSSKIIAKSVSLGDGKSIYRGLVRINKDAVNAKSHVQCDALLINDESVSYTMPHDEIYEPTA
hmu_Hmuk_2919 326 TIAMAGE.GQNIDTGAKVIHLAPNTKSTIESKSISKDGGRTNYRGLVHIADGAEDASTSVECDALMFDNESTSDTMPYMEIQESKV
3
mac_MA_4407 262 TIAIGHP.GSELDLGSRAVFNAPDTRAELISRTITI.GGRLVARG..EMIGNAKGAKGHLECKGLVLTDKGSQLAIPILEANVDDI
mac_MA_0936 200 TKVYGKA.DDKIKILERIALNGENARSVIKSRIAITDNAESVFRG..ITEGHAPRARGHVDCMEVIQG.DAKAEAVPIVRVDNPLA
mja_MJ_0034 194 TRTYAIK.EDVVKVNEVVKLNGENAKCIIKSRGAAMDNSKISLKL..KIEGNAPYSKGHIDCAEIVKG.NAEVESIPIVVRDDKA
afu_AF_2365 223 SVIVALE.GSVVDSGSRAVLMGENSRAEIISRTISK.GGKIIARG..HIIGDAPEVKGHLECKGLMLSESGLIDAIPELEARYPNV
mth_MTH_1150 264 SILGGQK.DSVLDMGSRVILEGRGSSAEMVSRAVSKDSSQIYSRG..HLAGRVPEVKGHLECHGLVLSDDSMIYAVPELEGSATEL
tpe_Tpen_1531 258 SVVAGTG.PGVYDVGGGAVLLGEGSSAELVSRVLGSKGSTIVSRG..SVEAEANDVKGHIECLGVMLDPSSTIEAVPVLKSRVERA
tag_Tagg_0540 215 STIGHRN..SRVLHNTRVRLKGSGSSAIVNVKSVAHDQSFIKHDIT..LEAWSGETKGHVECTGLLLG.NAVFETIPSLKSVSMDS
1
tko_TK0730 382 ELSHEAAIGKIREEELFYLMSRGLDEEKATQLIVKGFLDPMLKDIPMEFLVEIRKIIELAVSGGM...
ton_TON_0531 381 ELSHEAAIGKIREEELFYLMSRGLSEEKATQLIVKGFLEPMLKDIPMEFLVEIRKIIELAVSGGM...
pho_PH1385 382 ELSHEAAIGKIKEEELFYLMSRGLSEEKATQLIVKGFVEPMLKDIPIEFVVEIKKIIELAVSGGF...
abi_Aboo_0336 377 ELSHEAAIGKIREEELFYLMSRGLKEEEATQLIVKGFVNPLLKDIPMEFVIEIRKIIEMAVSGGM...
pfm_Pyrfu_1442 374 SFGHEASVGRLSAEKLYYLRAMGFEEDEATSLLIQGFFEPVFAGLPFDLAVEVRKIVELALRGH....
iho_Igni_1220 375 ELAHEGTVGKLGEEQLFYLRSMGFTEPEAVQMLTIGFIDPLVKDLPSDYVRAIREIVKMTLNAS....
2
ape_APE_1703 409 SVNHEATTGRLTEPQLFYLTSRGLTEEEAKSLIVLGFLEDVLKELPFEYANVLTKVVRLEFSEYGAFG
ttn_TTX_0943 405 VVTHEATASRISEEKLMYLRSRGFTEDEAKALVVLGFVSDIVKDLPFEYAVVLRRVLELEFSKLGAVG
pfu_PF1286 408 SIIHEATTGKLSEDKLFYLKARGIKEEEAKSLIVLGFISEVLEDLPFEYVEVLKKVIELEFSEIGGVG
sso_SSO0927 412 DVGHEAHTFRMNEDQLFYLMTRGIDEKEATSMLVLGFIDEIMKELPFEYATMLNKVIKLELDKLGAVA
tac_Ta0203 414 NFTHEATVGKIGTEELTYLRSRGLSEDEASSLIVLGFLDDVMKEIPMEFAVEMNRLVKLEMSKMGAVG
hmu_Hmuk_2919 411 DVAHEATVGKIGDEDVFYLQSRGLDDDDAKQMIVAGFIEPLTEELPIEYAVEMNRLIELEM..EGSLG
3
mac_MA_4407 344 ELTHEAAVGKIAKDQVEYLMARGLTEDEAVGMIIRGFLDVGIRGIPEELKKEIEETITQTALGM....
mac_MA_0936 282 KVTHEAAIGCVDKKEVETLMARGLEEDDAIDIIVKGMLA.............................
mja_MJ_0034 276 RITHEAAIGSVDKKQLETLMAKGLDEDEATEIIVKGMIGDL...........................
afu_AF_2365 305 ELSHEAAIGKIAEEGIFYLMSRGLSRDEAISAIVRGFMEIEIKGLPEALQEAIRRTIEMAERDLL...
mth_MTH_1150 347 EMSHEAAVGKIAEEEVMYLTSRGLTEEEAASMIVRGFLSMDITGLPEALAAETKRMLDMSLKGM....
tpe_Tpen_1531 341 ELTHEAAIGMLSGEKIEYLMARGFSEEEARSILLRGYLTAETPGLPESVKGEIERVIEYVVRHALG..
tag_Tagg_0540 296 ELTHEASLGKIKSDEVFYLMTRGLSEEEATRLIVVGFLSQVYEKLPKHLKEYLLSITRMFVSRTL...
B
1
tko_TK0730 1 MTETITMADAKAIIENQIEELARRNKEPEWMTKIRYKALEAFERAPHKDPVISEEQLLHFIAKPEVKGLPDHIESLD....DLPPE
ton_TON_0531 1 .MSEITIQEAKEIIAQEIENLARRNKEPEWMTRIRYKGLEAFEKAPHNDPVISKDELLRFIAKPEIEGLPEHIESLD....DLPPE
pho_PH1385 1 MSETLTLSDAKSIIENQIEELAKRNKEPEWMTKIRYKALEEFMKAPLNDPVIDEETLLNFIAKPEIEGIPEKVESLD....DLPPE
abi_Aboo_0336 1 .MSELTIQEARSIIEDQIEKLKKRNKEPEWMTKLRYKAMDAFFEAPHNDPVIKDP..LDYIAKTEEEMYPE.VKSLD....ELPPD
pfm_Pyrfu_1442 1 ...MTRLSEILGFSKRDLEEYARRRGEPEWLVRRRLEAYDALERLPPDPLIDEY...VKQLDLDAIFGFGGGPDIEVP.....KEY
iho_Igni_1220 1 ......MNEVQ.IIKYYAEEISKKLKEPEWALRLRLKAAELYPKVPAPPWMPE......DFDLEAFAAKAAEEEFVKPGEGEIPDW
3
mac_MA_4407 1 ........MQTDEMSLKKRAESAAEKKAAYGEDFELEKFEEGSK.VSKPIEDL..........................QTLDEE
mac_MA_0936 ....
mja_MJ_0034 ....
afu_AF_2365 1 .........MFGEMS.............GEYGELG..V
mth_MTH_1150 1 ........MLRDTLK...KAEKAREKKALYGEDIDLEKFIKEEAGEHEEVTRA..........................KEVPKE
tpe_Tpen_1531 1 .........MRLMERVEAARKALSKPSPYGPDLDLSSFRVAAP....GYSAG...........................EPPRAV
tag_Tagg_0540 1 ....MPSTDT
1
tko_TK0730 83 MKALLDRLGISEVEQKYIAGLAVQTDTGVIYNQFLQDWAKKGLIVLPTEEAVRKYPDIVKQHFLKLFRADESKLTAYHTAVWNG..
ton_TON_0531 82 MKALLDRLGISEVEQKYIAGLAVQTDTGIIYNQFLQEWAKKGLIVLPMEEAVKKYPDVVKRHFLQMFSVNESKMAAYHTAVWNG..
pho_PH1385 83 MKDLLDRLGINEVEQKYIAGLAVQTDTGVIYNQFLQEWAKKGLIVLPTEEAVRRYPDIMKEHFLKLFKAGESKLTAYHIAIWNG..
abi_Aboo_0336 79 MLALLDRLGIAEVEKKYIAGLAVQSDTSIVLNDFLKEWRKKGLIVESMEDAVRNHP.ILKDLFLKQFNPYESKLAAYHTAVWNG..
pfm_Pyrfu_1442 76 WDLAIKRLGIKPEELEALTGLAVTI.DNRVVEAQLRALQEKGVILEPMDEAVKKYD.WLKDYMLRIMRP.DNRHAAYHIMLWAG..
iho_Igni_1220 74 YKALLERIGVPDIEQSALAGVTVMVNENVVYEAQKRDLMRKGVILKSMDEAVREHEEFVKERLHSAQRPEAHKLAALHAALRRG..
3
mac_MA_4407 51 SKKTLLQVGII.PSEEGRSGSFIVLDNAVSHSSLK...DEN.VELMSTHKALEKYEWLKDYSWK.LVQVDADKYTAKTYLEDAD..
mac_MA_0936 1 MTQITLNALSRETEDITAAYTAAGGDAAVLHNHGLSSLVVSGNKVLSANATEGIVLEKKETENG......................
mja_MJ_0034 1 ....MSIKEELMEIIEAIKYTSEKPEEIVHGKGPR.IIVKESRIIDVQGDEGIILEGKEEDGK.......................
afu_AF_2365 15 DKERLKEVGIE.LDKSKRSGVYLQEDQDAKTFSSF...FEG.VEVMSIKQAMEKYDWVKDYFWK.ILRKDQDEFTRMADTEDVN..
mth_MTH_1150 49 VQETLLRVGVD.PEERERAGTFIQVDQDSGICTTCA...SES.IEIMGMNVALDKYSWLKDYMWK.AVAVDTDKYTATTALREAEGE
tpe_Tpen_1531 46 SSSVKERVGID.VSRASYVQVGETIFARVLAERLF...REYGVVVKPLSKALKEDGPAARVAWS.LIDPATDKYTAYAYLYGGEA.
tag_Tagg_0540 7 TDKLLAKTGLPFPGEVS....SVVVNEKLFMKSYIKTLSGKGIVLEDVKTAFNNKEYAEKIINI..LEKNGLKKDVFQQQGLTG..

C

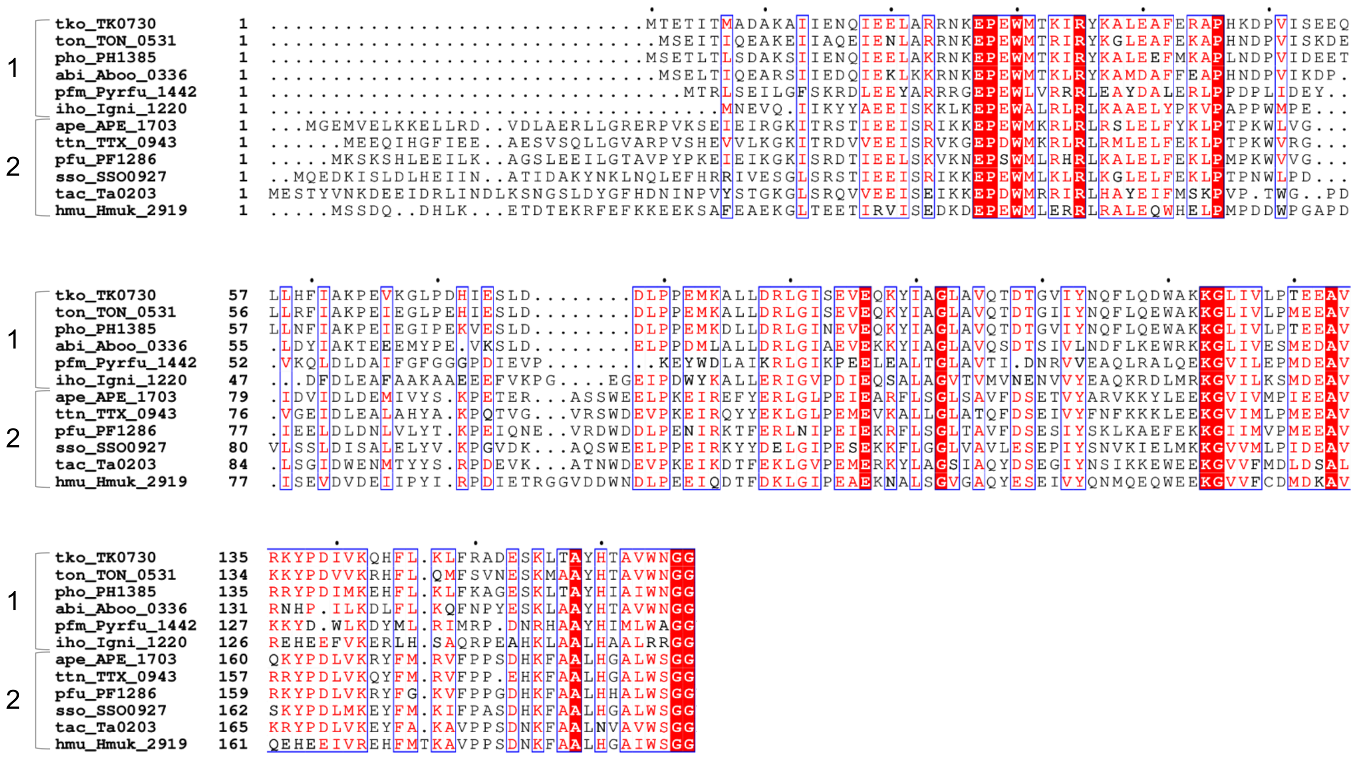

Fig. S7. Amino acid sequence alignment of SufB and SmsB homologs. (A) Amino acid sequence alignment of the SmsB homologs, SmsB proteins in a clade including *Tk*-SmsB (group 1), SufB proteins (group 2), and SmsB proteins in a clade including methanogenic SmsBs (group 3). The N-terminal sections are indicated by a black bar. (B) Sequence alignment of the proteins in group 1 and 3. (C) Sequence alignment of the proteins in group 1 and 2. In panels (B) and (C), only the N-terminal regions of the sequences are shown. The names of the sequences are their locus tags, consistent with those in Fig. 1. Similar residues in the columns are shown by red letters, and identical residues are shown by white letters on a red background. The columns that display high similarity (global score higher than 0.7) are framed in blue.

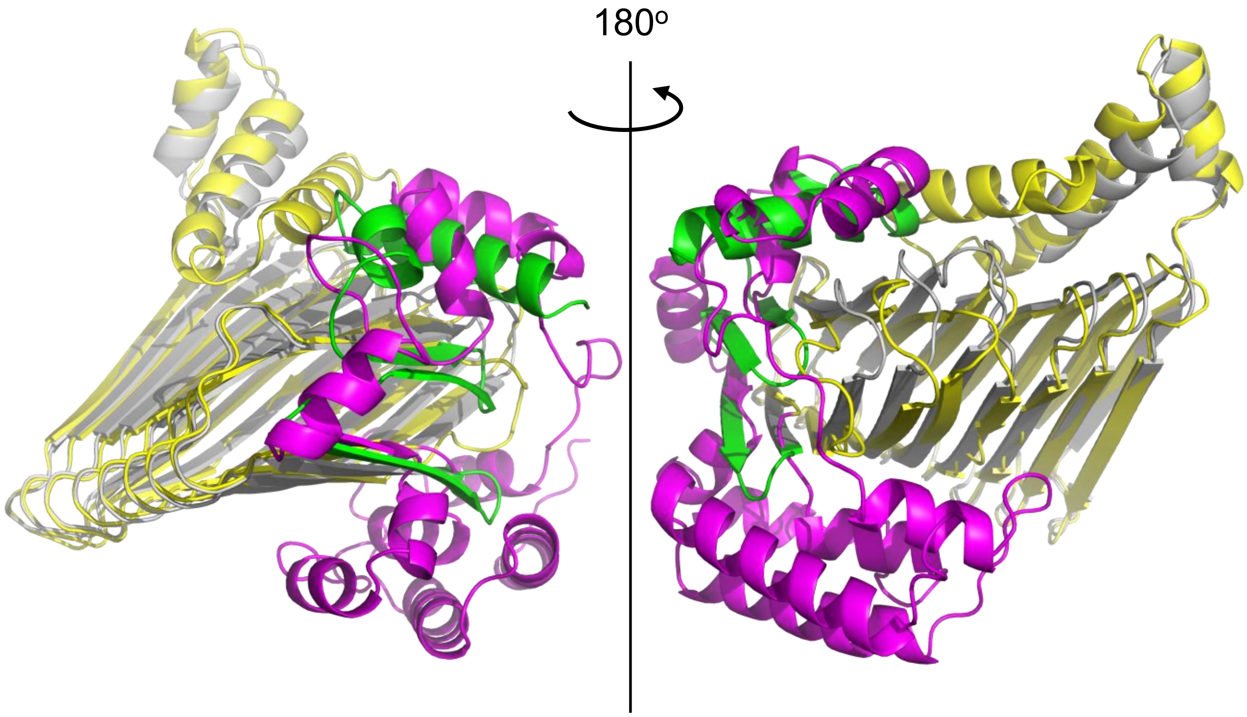

Fig. S8. Structural comparison of *Tk*-SmsB and methanogenic SmsB. Structural superimposition of *Tk*-SmsB (yellow) with SmsB from *M. jannaschii* (MJ_0034, *Mj*-SmsB; gray). The structure of *Mj*-SmsB protein (AF-Q60349-F1-v4) was retrieved from the AlphaFold Protein Structure Database. The N-terminal sections of *Tk*-SmsB and *Mj*-SmsB are colored by magenta and green, respectively.
